# Clinical Benefits of Combination Immunotherapy Over Standard Immunotherapy Monotherapy in Previously Treated Advanced Esophageal Squamous Cell Carcinoma: A Systematic Review and Meta‐Analysis

**DOI:** 10.1002/cam4.71329

**Published:** 2025-10-29

**Authors:** Yong Chen, Zixuan Chen, Hong Guo, Heng Zhou, Yizhou Deng, Rui Wang, Fang Han, Haiyan Mao, Zhengrong Zhang, Yunjiang Wu, Ying Li

**Affiliations:** ^1^ Department of Radio‐Chemotherapy Affiliated Hospital of Yangzhou University, Yangzhou University Yangzhou City Jiangsu Province China; ^2^ Department of Thoracic Surgery Affiliated Hospital of Yangzhou University, Yangzhou University Yangzhou City Jiangsu Province China; ^3^ School of Public Health Yangzhou University Yangzhou City Jiangsu Province China

**Keywords:** combination immunotherapy, esophageal squamous cell cancer, PD‐1 inhibitor, second‐line therapy

## Abstract

**Purpose:**

Programmed cell death protein 1 (PD‐1) inhibitor monotherapy is the standard second‐line treatment for esophageal squamous cell carcinoma (ESCC), but the clinical response and survival outcomes still remain unsatisfactory. This systematic review aims to assess the efficacy and safety of combined immunotherapy strategies in previously treated ESCC patients.

**Methods and Materials:**

Studies involving previously treated ESCC patients treated with either combined immunotherapy or PD‐1 inhibitor monotherapy as second‐ or later‐line treatment were searched up to November 30, 2023. Pooled rates of objective response rate (ORR), disease control rate (DCR), progression‐free survival (PFS), overall survival (OS), and treatment‐related adverse events (TRAEs) were compared.

**Results:**

A total of 19 studies involving 3007 ESCC patients were included in the pooled analysis. Among them, 1308 patients received immunotherapy monotherapy. Combination immunotherapy included PD‐1 inhibitor combined with chemotherapy (123 patients), anti‐angiogenesis therapy (291 patients), chemoradiotherapy (49 patients), TIGIT inhibitor (62 patients), and anti‐EGFR antibody (28 patients). Patients receiving combination immunotherapy had significantly higher ORR, DCR, PFS, and OS rates compared to those receiving PD‐1 inhibitor monotherapy or chemotherapy (ORR: 35.5% vs. 19.8% vs. 13.0%, *p* = 0.000; DCR: 84.8% vs. 51.2% vs. 55.2%, *p* = 0.000). Subgroup analysis demonstrated that second‐line combination immunotherapy significantly improved response and survival rates compared to PD‐1 inhibitor monotherapy in immunotherapy‐naive ESCC patients. The limited data showed that PD‐1 inhibitors combined with both anti‐angiogenesis agents and chemotherapy as second‐line therapy improved response and survival rates compared to PD‐1 inhibitor monotherapy. Notably, the PD‐1 inhibitor combined with anti‐angiogenesis therapy or chemotherapy also showed high antitumor activity in immunotherapy‐treated ESCC patients. Combination therapy was associated with higher treatment‐related but manageable toxicity compared with PD‐1 inhibitor monotherapy.

**Conclusions:**

Based on the limited data, combined immunotherapy provides additional clinical benefits over standard PD‐1 inhibitor monotherapy in second‐line treatments for both immunotherapy‐naive and previously immunotherapy‐treated ESCC patients.

## 1 Introduction

Esophageal cancer (EC) is a prevalent malignancy worldwide, ranking 11th in incidence and 7th in cancer‐related deaths, with an estimated 511,000 new cases and 445,000 deaths globally in 2022 [1]. Approximately 75% of all new cases and deaths occurred in Asia [2]. In China, it was estimated that EC ranks 6th in cancer incidence and 4th in cancer death, with an estimated 346,633 new cases and 323,600 deaths in 2022 [3]. Eastern Asia has the highest regional incidence rates of EC, largely due to China's significant disease burden, with over half of the world's new cases and deaths from EC occurring there [4]. Clinically, over one‐third of patients are diagnosed at an advanced stage with distant metastasis, and the 5‐year survival rate is only 6% for advanced stages and 21% across all stages in the USA [5]. Due to continuous advancements in multidisciplinary treatments, the 5‐year survival rate for EC in China has improved from 27.8% (2008–2009) to 33.4% (2015–2017) [6].

Among all histological types of EC, esophageal squamous cell carcinoma (ESCC) accounts for up to 85.8% of EC cases in China [1]. With the advent of immune checkpoint inhibitors (ICIs), the treatment landscape for ESCC has changed significantly. Recently, the potential of neoadjuvant immunotherapy for resectable ESCC has been reported, offering promising new avenues for treatment [7]. For advanced and metastatic ESCC, a new standard of treatment has evolved throughout the disease process based on a series of randomized clinical trials since the implementation of ICIs, including PD‐1/programmed death ligand 1 (PD‐L1) inhibitors and cytotoxic T‐lymphocyte‐associated protein 4 (CTLA‐4) inhibitors. These agents have shown improved treatment efficacy and prolonged survival compared to conventional chemotherapy in either first‐ or second‐line treatment of ESCC [8, 9, 10, 11, 12, 13, 14, 15, 16, 17]. Phase III trials consistently demonstrated that combining anti‐PD‐1/PD‐L1 antibodies with conventional chemotherapy as a first‐line treatment for unresectable, locally advanced, recurrent, or metastatic ESCC significantly improved treatment efficacy and prognosis compared to chemotherapy alone, with an absolute increase in objective response rate (ORR) from 10.0% to 21.1% and an extension in median overall survival (mOS) by 2.8–6.6 months [10, 11, 12, 13, 14, 15, 16, 17]. These results ultimately changed clinical practice and have been recommended by current guidelines from the European Society for Medical Oncology (ESMO), National Comprehensive Cancer Network (NCCN), and Chinese Society of Clinical Oncology (CSCO).

For second‐line treatment of EC, results from KEYNOTE‐181 showed that pembrolizumab significantly improved the ORR (21.5% vs. 6.1%), mOS (9.3 vs. 6.7 months), and 1‐year OS rate (43.0% vs. 20.4%) compared to chemotherapy in EC patients with PD‐L1 combined positive score (CPS) ≥ 10 [18]. Among ESCC patients with PD‐L1 CPS ≥ 10, mOS was 10.3 months for pembrolizumab compared to 6.7 months for chemotherapy [18]. In 2019, the Food and Drug Administration (FDA) first approved pembrolizumab for patients with PD‐L1 expression (CPS ≥ 10) based on the results of the KEYNOTE‐180 and KEYNOTE‐181 trials [18, 19]. Subsequently, nivolumab and tislelizumab were recommended for second‐line treatment in the NCCN guideline, following the ATTRACTION‐3 and RATIONALE 302 trials [20, 21]. In addition to pembrolizumab, nivolumab, and tislelizumab, camrelizumab was also approved for second‐line treatment of ESCC in the CSCO guideline, based on the ESCORT trial [22]. Conventional chemotherapy options, including single‐agent therapies (docetaxel, paclitaxel, and irinotecan) and combination regimens (FOLFIRI, irinotecan plus cisplatin, and irinotecan plus docetaxel), remain viable alternatives for second‐line or subsequent therapy in ESCC. Thus, PD‐1 inhibitor monotherapy or chemotherapy remains the standard for second‐line or subsequent treatment of ESCC, with no significant changes to the guideline since 2019. A meta‐analysis by Jin et al. [8] demonstrated significantly higher ORR in the PD‐1 inhibitor group (Risk ratio (RR) = 1.88, *p* < 0.001) and better OS (Hazard ratio (HR) = 0.73, *p* < 0.001) compared to standard chemotherapy. However, the ORR in the PD‐1 inhibitor group was still relatively low, ranging from 12.8% to 20.3%, with mOS between 7.2 and 10.9 months. Due to these limited outcomes, the second‐line treatment strategy for ESCC urgently needs further optimizations to improve patient prognosis.

In the real‐world clinical practice, there are many rational combination strategies of PD‐1 inhibitor with other therapies, including chemotherapy, anti‐angiogenesis therapy, radiotherapy, immunomodulator drugs, and intratumoural therapies [23, 24]. In the CAP 02 trial (NCT03736863), Meng et al. reported that for unresectable locally advanced, recurrent, or metastatic ESCC patients who had progressed after or were intolerant to first‐line chemotherapy, treatment with camrelizumab combined with apatinib achieved a confirmed ORR of 34.6% (18/52). The median progression‐free survival (mPFS) was 6.8 months, and the mOS was 15.8 months. Notably, the treatment efficacy was maintained regardless of PD‐L1 expression [25]. A retrospective study conducted by Yang et al. demonstrated that PD‐1 inhibitor‐based combination therapy (either with anti‐angiogenesis therapy or chemotherapy) significantly improved outcomes compared to PD‐1 inhibitor monotherapy in patients with recurrent or metastatic advanced ESCC who progressed after first‐line chemotherapy (mPFS: 8.5 months vs. 3.2 months, *p* < 0.001; mOS: 18.9 months vs. 9.8 months, *p* = 0.010) [26]. Preliminary results from the CAP 02 Rechallenge study also showed that second‐line treatment with camrelizumab plus apatinib achieved an ORR of 36.8% and a disease control rate (DCR) of 89.5% in immunochemotherapy‐treated advanced ESCC patients [27]. With increasing clinical use of PD‐1/PD‐L1 inhibitor combination therapies for second‐line ESCC treatment, there is a strong need for a systematic analysis to provide more robust evidence supporting this combination strategy in clinical practice.

In the present study, we conducted a systematic review and meta‐analysis to evaluate the clinical efficacy and safety of combined immunotherapy as a second‐ or later‐line treatment for advanced ESCC.

## 2 Methods and Materials

### 2.1 Search Strategy and Study Selection

Relevant studies were retrieved from Web of Science, Embase, the Cochrane Library, Medline, and using MeSH synonyms, keywords, and free‐text searches. The search strategy is detailed in Table S1. The literature search was conducted without restrictions on language or publication date, with the final search deadline set for November 30, 2023. References and citations of the included studies were manually screened to identify additional relevant studies. When multiple publications were available for the same trial, the most recent version was selected for inclusion.

### 2.2 Inclusion and Exclusion Criteria

Studies eligible for the analysis met the following criteria: (1) Patients with unresectable, advanced, or metastatic ESCC confirmed histologically or cytologically; (2) Progressed to or intolerant to first‐line antitumor therapy, including either chemotherapy (immunotherapy‐naive) or PD‐1/PD‐L1 inhibitors (immunotherapy‐treated); (3) Prospective or retrospective study; (4) Patients receiving PD‐1/PD‐L1 inhibitor‐based combined therapy (additional radiotherapy was acceptable) as second‐ or later‐line treatment; and (5) At least one endpoint, such as OS, PFS, ORR, DCR, and TRAEs can be extracted. The exclusion criteria for studies are as follows: (1) Studies that included patients with adenocarcinomas of the esophagus or gastroesophageal junction, where the outcomes for ESCC patients could not be extracted separately; (2) Data cannot be extracted from the publication; (3) Studies based on cancer databases; (4) Summative articles such as review articles, meta‐analyses, systematic reviews, or editorial comments.

### 2.3 Data Extraction

Data were extracted independently by two investigators, with the primary endpoints including ORR, DCR, PFS, OS, and TRAEs. Any disagreements were resolved by consensus with a third investigator. Kaplan–Meier curves were digitized using Engauge Digitizer (version 4.1).

### 2.4 Quality Assessment

The Cochrane risk of bias tool was used to assess the quality of randomized controlled trials (RCTs) [28], the Case Series Reporting Bias Evaluation Tool was applied for quality assessment of single‐arm studies (Available at: http://www.ihe.ca/advanced‐search/development‐of‐a‐quality‐appraisal‐tool‐for‐case‐series‐studies‐using‐a‐modified‐delphi‐technique, accessed Sep 01, 2024), and the Newcastle‐Ottawa Quality Assessment Scale was used to evaluate the quality of cohort studies [29].

### 2.5 Statistical Analysis

R Studio software (version 4.3.3) was used to perform the analyses and to draw forest plots for the pooled analyses. Nonnormally distributed data were subjected to a Freeman‐Tukey double‐anti‐sine transformation prior to analysis and then merged into a single rate using the Metaprop command. Review Manager 5.3 was used for meta‐analysis. Heterogeneity‐related variation in effectiveness estimates across studies was assessed using *I* ^2^, which spans from 0% (meaning all heterogeneity is artificial) to 100% (meaning all heterogeneity is actual). When heterogeneity was observed (*I* ^2^ > 50%), a random effects model was used; conversely, a fixed effects model was used. Potential bias was investigated using funnel plots and Egger's bias test. The trim‐and‐fill method was used to evaluate the influence of potential publication bias. All *p*‐values were two‐sided and *p*‐values < 0.05 were considered statistically significant.

## 3 Results

### 3.1 Literature Search and Description of the Studies

**FIGURE 1:**
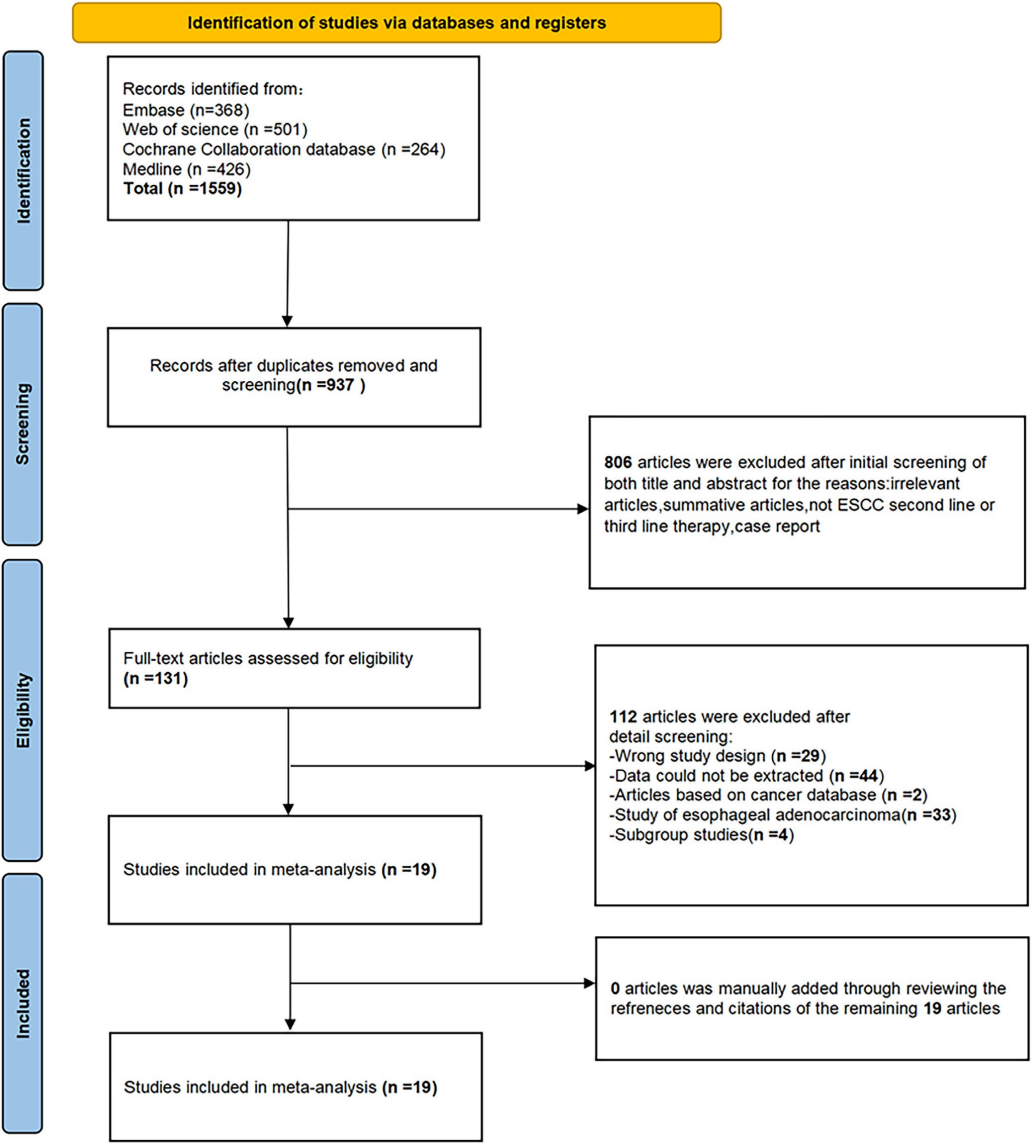
PRISMA flow chart. PRISMA, Preferred Reporting Items for Systematic Reviews and Meta‐Analyses.

The flowchart of the study search and screening process is shown in Figure 1. A total of 1559 publications were initially identified. After multiple stages of screening, 19 articles involving 3007 ESCC patients were ultimately included in the analysis. Details for each study were shown in Table S2. Five studies included patients who received third‐ or later‐line immunotherapy, while the remaining studies focused on patients treated with second‐line immunotherapy. Of these, 569 patients received PD‐1 inhibitor‐based combination immunotherapy (123 with chemotherapy, 291 with anti‐angiogenesis therapy, 16 with anti‐angiogenesis therapy plus chemotherapy, 49 with radiotherapy plus chemotherapy, 62 with TIGIT inhibitor, and 28 with anti‐EGFR antibody); 1308 patients were treated with PD‐1 inhibitor monotherapy, and 1130 patients received chemotherapy.

### 3.2 Quality Assessment and Publication Bias

**FIGURE 2:**
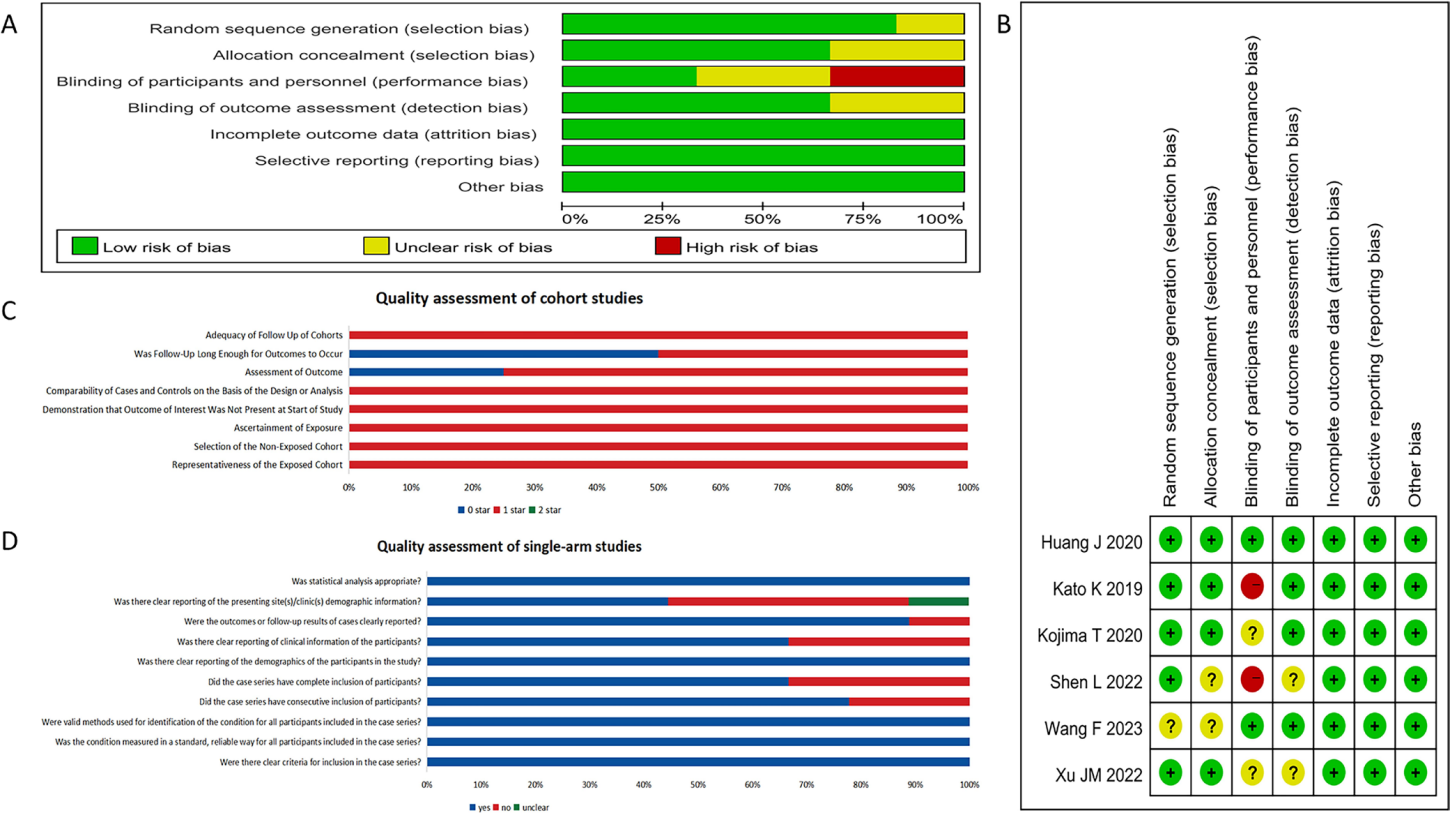
Literature quality evaluation. Cochrane reviews for randomized controlled trials: Risk of bias graph (A) and risk of bias summary (B). Quality evaluation of cohort studies (C). Quality evaluation of single‐arm studies (D).

Among the 19 studies, six were RCTs, four were cohort studies, and nine were single‐arm studies. Quality evaluation for included studies is shown in Figure 2. Two RCTs had a high risk of selection and performance bias (Figure 2A,B, Table S3). The quality of all the four cohort studies was moderate to high, with scores ranging from 6 to 8 (median score 7.5, Figure 2C, Table S4). Most single‐arm studies were assessed as having a low risk of bias (Figure 2D, Table S5). Publication bias may be present in the results of DCR, 3m‐, and 6m‐PFS rates based on the funnel plot and Egger's test (Figures S1 and S2). However, these values remained unchanged after applying the trim and fill method (Figure S1K–M).

### 3.3 Clinical Benefit of PD‐1 Inhibitor Monotherapy as Second‐ or Later‐Line Treatment for ESCC

**FIGURE 3:**
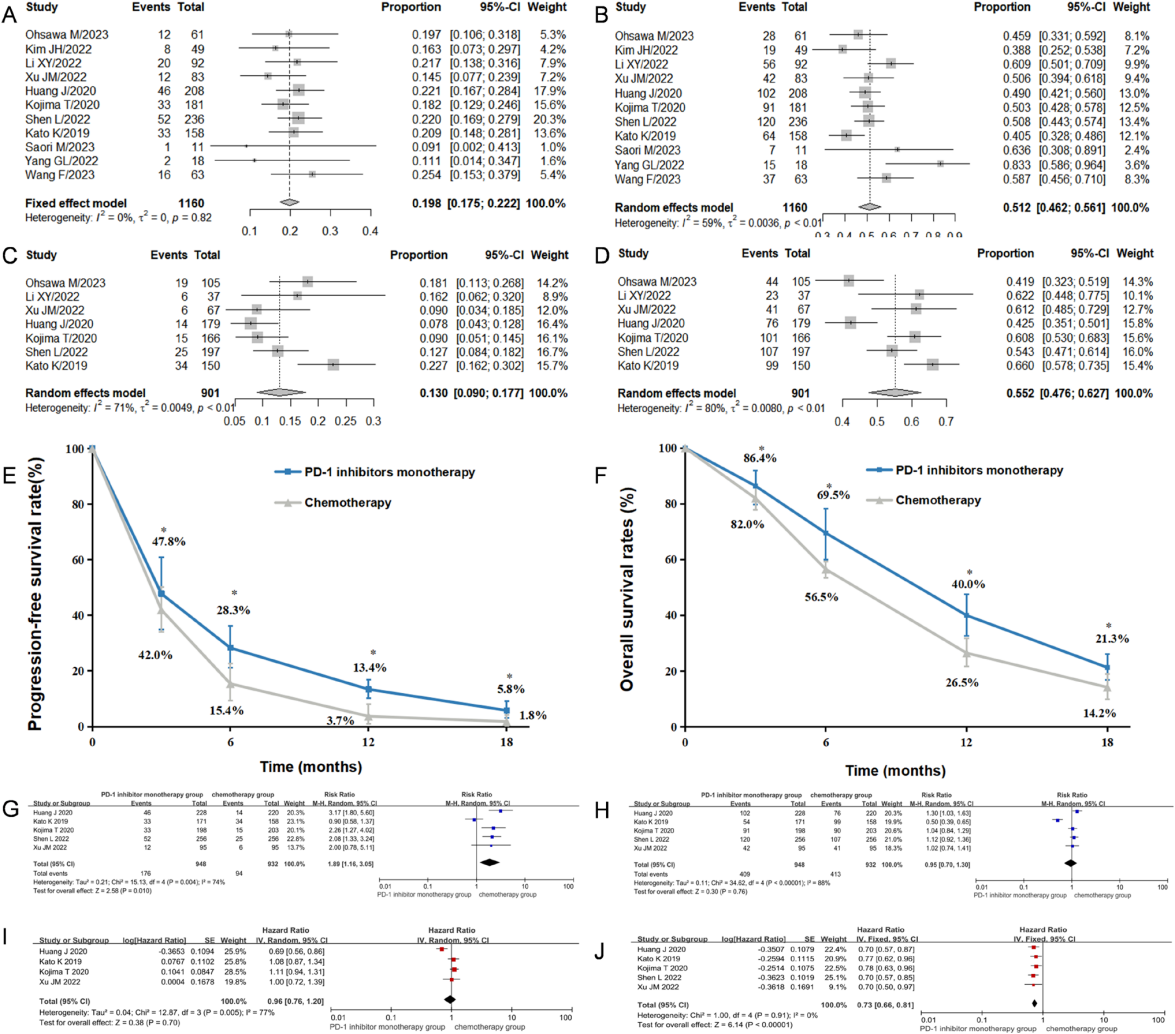
Clinical treatment efficacy of PD‐1 inhibitor monotherapy and chemotherapy in ESCC patients. Forest plots of ORR (A) and DCR (B) for patients treated with second or later‐line PD‐1 inhibitor monotherapy and forest plots of ORR (C) and DCR (D) for patients treated with second or later‐line chemotherapy. Pooled PFS rates (E) and OS rates (F) for the second or later‐line PD‐1 inhibitor monotherapy group and chemotherapy group. Forest plots of ORR (G), DCR (H), PFS (I), and OS (J) in ESCC patients treated with second‐line PD‐1 inhibitor monotherapy or chemotherapy through meta‐analysis of RCTs. DCR, Disease control rate; ESCC, Esophageal squamous cell carcinoma; ORR, Objective response rate; OS, Overall survival; PD‐1, Programmed cell death protein 1; PFS, Progression‐free survival; RCTs, Randomized controlled trials; *, *p* < 0.05.

A total of six RCTs, three cohort studies, and two single‐arm studies were included in this analysis. The pooled analysis revealed that the PD‐1 inhibitor monotherapy group had a significantly higher ORR compared to the chemotherapy group (19.8% [95% CI: 0.075–0.222, *I* ^2^ = 0%] vs. 13.0% [95% CI: 0.090–0.177, *I* ^2^ = 71%], *p* = 0.000, Figure 3A,C), while the DCR was similar between the two groups (51.2% [95% CI: 0.462–0.561, *I* ^2^ = 59%] vs. 55.2% [95% CI: 0.476–0.627, *I* ^2^ = 80%], *p* = 0.074, Figure 3B,D). In the PD‐1 inhibitor monotherapy group, the median follow‐up time of the included studies ranged from 7.1 to 16.0 months. The pooled mPFS was 2.5 months (range, 1.6–7.4 months), and the pooled mOS was 9.2 months (range, 6.4–14.1 months). The pooled 3‐, 6‐, 12‐, and 18‐month PFS rates were 47.8% [95% CI: 0.349–0.608, *I* ^2^ = 95%], 28.3% [95% CI: 0.212–0.361, *I* ^2^ = 87%], 13.4% [95% CI: 0.103–0.168, *I* ^2^ = 57%], and 5.8% [95% CI: 0.031–0.091, *I* ^2^ = 76%], respectively (Figure S3A–D). The pooled 3‐, 6‐, 12‐, and 18‐month OS rates were 86.4% [95% CI: 0.797–0.920, *I* ^2^ = 89%], 69.5% [95% CI: 0.599–0.783, *I* ^2^ = 91%], 40.0% [95% CI: 0.333–0.475, *I* ^2^ = 84%], and 21.3% [95% CI: 0.169–0.262, *I* ^2^ = 71%], respectively (Figure S3E–I). In the chemotherapy group, the median follow‐up time of the included studies ranged from 5.8 to 14.1 months. The pooled mPFS and mOS were 2.8 months (range, 1.9–7.1 months) and 7.0 months (range, 6.2–8.4 months), respectively. The pooled 3‐, 6‐, 12‐, and 18‐month PFS rates were 42.0% [95% CI: 0.341–0.501, *I* ^2^ = 86%], 15.4% [95% CI: 0.094–0.226, *I* ^2^ = 89%], 3.7% [95% CI: 0.009–0.081, *I* ^2^ = 89%], and 1.8% [95% CI: 0.004–0.042, *I* ^2^ = 80%], respectively (Figure S4A–D). The pooled 3‐, 6‐, 12‐, and 18‐month OS rates were 82.0% [95% CI: 0.778–0.860, *I* ^2^ = 68%], 56.5% [95% CI: 0.535–0.594, *I* ^2^ = 31%], 26.5% [95% CI: 0.217–0.317, *I* ^2^ = 71%], and 14.2% [95% CI: 0.100–0.191, *I* ^2^ = 78%], respectively (Figure S4E–I). The PFS rates, as well as the OS rates, were significantly higher in the PD‐1 inhibitor monotherapy group compared to the chemotherapy group (Figure 3E,F).

After excluding two studies involving patients who received third‐ or later‐line immunotherapy, a total of nine studies were included to analyze the difference in second‐line treatment efficacy between PD‐1 inhibitor monotherapy and chemotherapy. The results showed that the pooled ORR, DCR, PFS, and OS rates were significantly higher in the PD‐1 inhibitor monotherapy group compared to the chemotherapy group (Figures S5 and S6). We further refined our meta‐analysis to 5 RCTs to avoid publication bias, and our results again showed a significant benefit of ORR and OS in the second‐line PD‐1 inhibitor monotherapy group as compared with the second‐line chemotherapy group (ORR: RR = 1.89, 95% CI: 1.16–3.05, *I* ^2^ = 74%, *p* = 0.01; OS: HR = 0.73, 95% CI: 0.66–0.81, *I* ^2^ = 0%, *p* < 0.001) (Figure 3G–J).

### 3.4 Clinical Efficacy of Combination Immunotherapy as Second or Later‐Line Treatment in ESCC Patients

**FIGURE 4:**
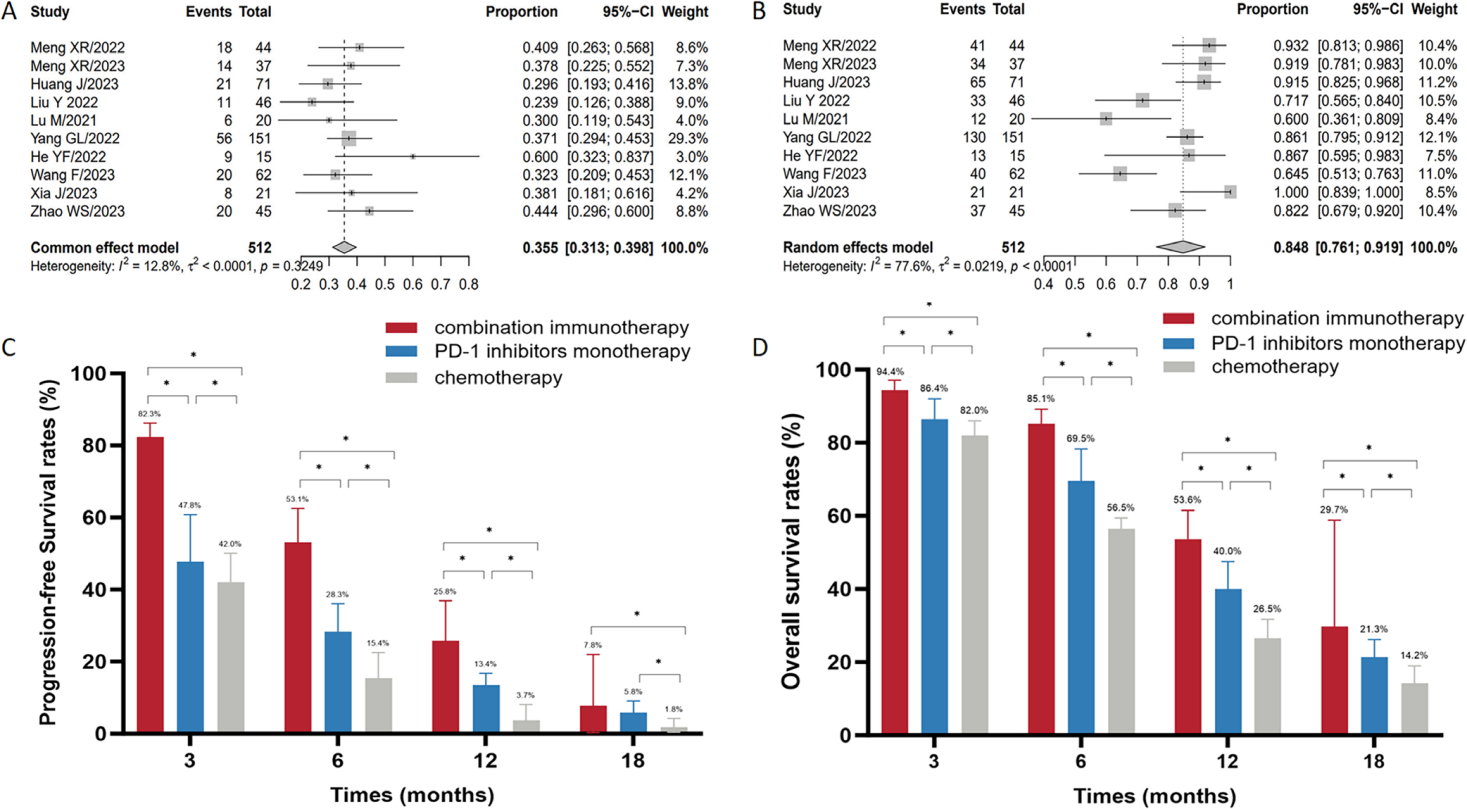
Clinical benefits of second or later‐line combination immunotherapy in ESCC patients. Forest plots of ORR (A) and DCR (B) for patients treated with combination immunotherapy. Pooled analysis of PFS (C) and OS (D) rates among the combination immunotherapy, PD‐1 inhibitor monotherapy, and chemotherapy groups. DCR, Disease control rate; ESCC, Esophageal squamous cell carcinoma; ORR, Objective response rate; OS, Overall survival; PD‐1, Programmed cell death protein 1; PFS, Progression‐free survival; *, *p* < 0.05.

A total of 10 studies (one RCT, two cohort studies, and seven single‐arm studies) were included in this analysis. These combinations included PD‐1 inhibitor combined with anti‐angiogenesis therapy, chemotherapy, chemoradiotherapy, TIGIT, or EGFR inhibitors. Both immunochemotherapy‐treated and immunochemotherapy‐naive patients were included in the analysis. The pooled ORR and DCR were 35.5% [95% CI: 0.313–0.398, *I* ^2^ = 13%] and 84.8% [95% CI: 0.768–0.914, *I* ^2^ = 78%], respectively (Figure 4A,B). Pooled analysis revealed that the combination immunotherapy had a significantly higher ORR and DCR compared to both the PD‐1 inhibitor monotherapy and chemotherapy (ORR: 35.5% [95% CI: 0.313–0.398, *I* ^2^ = 13%] vs. 19.8% [95% CI: 0.075–0.222, *I* ^2^ = 0%] vs. 13.0% [95% CI: 0.090–0.177, *I* ^2^ = 71%], *p* = 0.000; DCR: 84.8% [95% CI: 0.768–0.914, *I* ^2^ = 78%] vs. 51.2% [95% CI: 0.462–0.561, *I* ^2^ = 59%] vs. 55.2% [95% CI: 0.476–0.627, *I* ^2^ = 80%], *p* = 0.000). The median follow‐up time of the included studies with combination immunotherapy ranged from 7.5 to 14.0 months. The pooled mPFS and mOS were 6.4 months (range, 2.7–10.9 months) and 13.7 months (range, 7.5–19.3 months), respectively. The pooled 3‐, 6‐, 12‐, and 18‐month PFS rates were 82.3% [95% CI: 0.780–0.862, *I* ^2^ = 0%], 53.1% [95% CI: 0.435–0.625, *I* ^2^ = 67%], 25.8% [95% CI: 0.160–0.369, *I* ^2^ = 79%], and 7.8% [95% CI: 0.003–0.220, *I* ^2^ = 92%], respectively (Figure S7A–D). The pooled 3‐, 6‐, 12‐, and 18‐month OS rates were 94.4% [95% CI: 0.911–0.971, *I* ^2^ = 5%], 85.1% [95% CI: 0.804–0.892, *I* ^2^ = 0%], 53.6% [95% CI: 0.456–0.615, *I* ^2^ = 52%], and 29.7% [95% CI: 0.072–0.588, *I* ^2^ = 95%], respectively (Figure S7E–H). The pooled FPS and OS rates (except for the 18‐month PFS rate) were significantly higher in the combination immunotherapy group than in the PD‐1 inhibitors monotherapy group (Figure 4C,D).

### 3.5 Combination Immunotherapy Demonstrated High Treatment Efficacy in Immunochemotherapy‐Treated Advanced ESCC Patients

There were three studies reported on the treatment efficacy of combination immunotherapy in advanced ESCC patients who progressed prior to immunotherapy (PD‐1 inhibitor combined with anti‐angiogenesis therapy: two studies; PD‐1 inhibitor combined with chemoradiotherapy: one study). The pooled ORR and DCR were 35.8% [95% CI: 0.283–0.437, *I* ^2^ = 26%] and 89.3% [95% CI: 0.836–0.939, *I* ^2^ = 19%], respectively. The pooled mPFS and mOS were 6.0 months (range, 4.6–6.9 months) and 10.6 months (range, 7.5–12.8 months), respectively. The pooled 3‐, 6‐, 12‐, and 18‐month PFS rates were 77.7% [95% CI: 0.688–0.855, *I* ^2^ = 0%], 45.8% [95% CI: 0.247–0.677, *I* ^2^ = 80%], 21.9% [95% CI: 0.082–0.396, *I* ^2^ = 73%], and 8.4% [95% CI: 0–0.489, *I* ^2^ = 96%], respectively. Due to the limited data, the pooled 12‐ and 24‐month OS rates were 47.6% [95% CI: 0.394–0.558, *I* ^2^ = 0%] and 27.5% [95% CI: 0.205–0.352, *I* ^2^ = 0%], respectively (Figure S8).

### 3.6 Clinical Efficacy of Second‐Line Combination Immunotherapy in Immunotherapy‐Naive ESCC Patients

**FIGURE 5:**
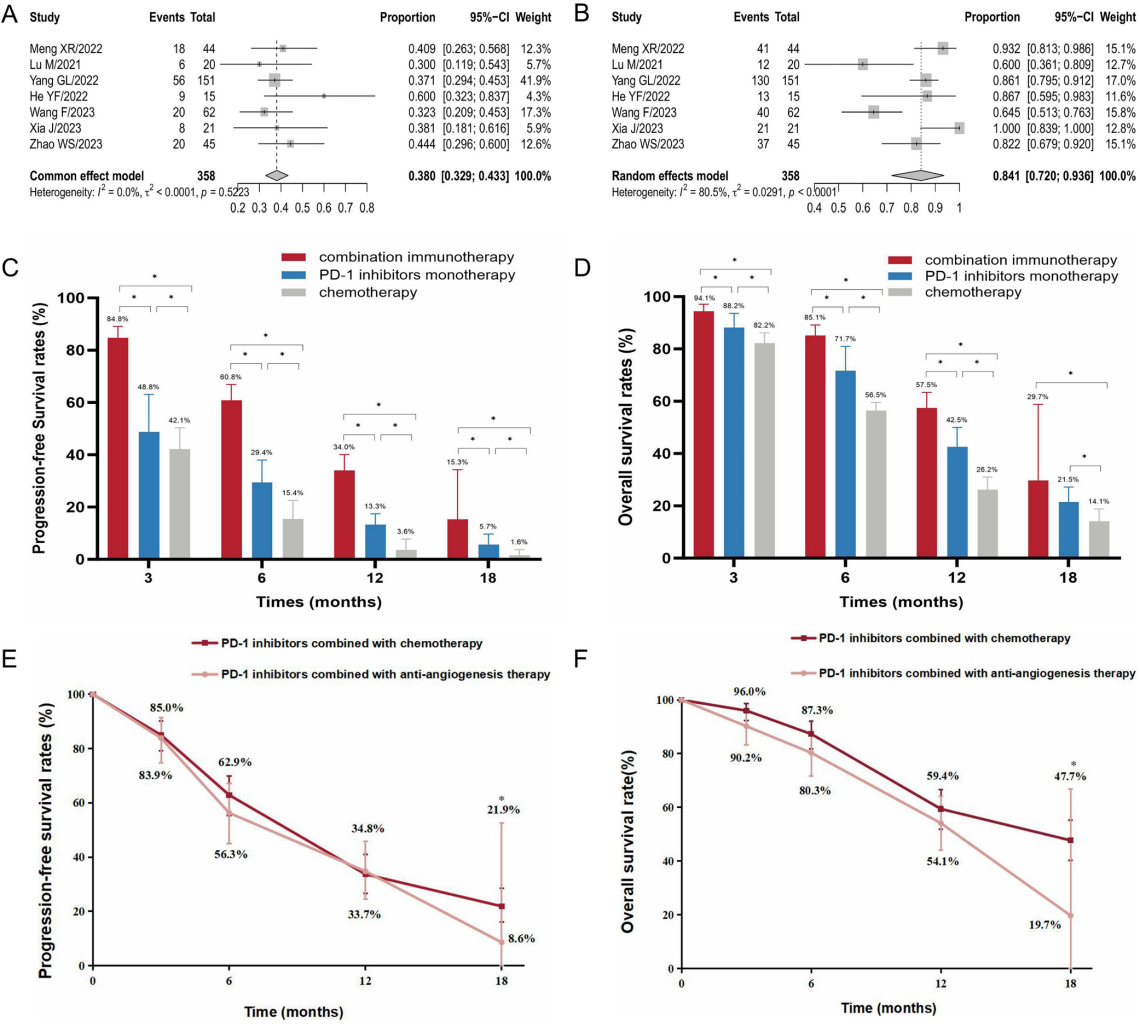
Clinical efficacy of second‐line combination immunotherapy in immunotherapy‐naive ESCC patients. Forest plots of ORR (A) and DCR (B) for patients treated with second‐line combination immunotherapy. Pooled analysis of PFS (C) and OS (D) rates among the combination immunotherapy, PD‐1 inhibitor monotherapy, and chemotherapy groups. Pooled analysis of PFS (E) and OS (F) rates in PD‐1 inhibitor combined with anti‐angiogenesis therapy or chemotherapy. DCR, Disease control rate; ESCC, Esophageal squamous cell carcinoma; ORR, Objective response rate; OS, Overall survival; PD‐1, Programmed cell death protein 1; PFS, Progression‐free survival; *, *p* < 0.05.

Since the PD‐1 inhibitor monotherapy is currently the standard treatment for the ESCC patients, the present study further analyzed the clinical efficacy of second‐line combination immunotherapy in immunotherapy‐naive ESCC patients. A total of seven studies were included in this analysis. The combination strategies included PD‐1 inhibitor combined with anti‐angiogenesis therapy (three studies), chemotherapy (two studies including one study combined with chemoradiotherapy), anti‐angiogenesis therapy plus chemotherapy (one study), TIGIT (one study), or EGFR inhibitors (one study). The pooled ORR and DCR were 38.0% [95% CI: 0.329–0.433, *I* ^2^ = 0%] and 84.1% [95% CI: 0.732–0.928, *I* ^2^ = 80%], respectively (Figure 5A,B). Pooled analysis revealed that second‐line combination immunotherapy had a significantly higher ORR and DCR compared to the PD‐1 inhibitor monotherapy (ORR: 38.0% [95% CI: 0.329–0.433, *I* ^2^ = 0%] vs. 20.6% [95% CI: 0.182–0.231, *I* ^2^ = 0%], *p* = 0.000; DCR: 84.1% [95% CI: 0.732–0.928, *I* ^2^ = 80%] vs. 52.7% [95% CI: 0.474–0.580, *I* ^2^ = 62%], *p* = 0.000). The median follow‐up time of the included studies with second‐line combination immunotherapy ranged from 7.5 to 14.0 months. The pooled mPFS and mOS were 6.8 months (range, 2.7–10.9 months) and 15.4 months (range, 10.1–19.3 months), respectively. The pooled 3‐, 6‐, 12‐, and 18‐month PFS rates were 84.8% [95% CI: 0.799–0.891, *I* ^2^ = 0%], 60.8% [95% CI: 0.546–0.669, *I* ^2^ = 0%], 34.0% [95% CI: 0.282–0.401, *I* ^2^ = 0%], and 15.3% [95% CI: 0.029–0.343, *I* ^2^ = 91%], respectively (Figure S9A–D). The pooled 3‐, 6‐, 12‐, and 18‐month OS rates were 94.4% [95% CI: 0.911–0.971, *I* ^2^ = 5%], 85.1% [95% CI: 0.804–0.892, *I* ^2^ = 0%], 57.5% [95% CI: 0.514–0.634, *I* ^2^ = 40%], and 29.7% [95% CI: 0.072–0.588, *I* ^2^ = 95%], respectively (Figure S9E–H). The pooled FPS and OS rates from the combination immunotherapy group were significantly higher than those from the PD‐1 inhibitor monotherapy group in immunotherapy‐naive ESCC patients (Figure 5C,D).

Since the PD‐1 inhibitor combined with anti‐angiogenesis or chemotherapy was the main second‐line combination strategy in immunotherapy‐naive ESCC patients, the current study attempted to explore the benefits of the second‐line PD‐1 inhibitor combined with anti‐angiogenesis as compared with chemotherapy. In the PD‐1 inhibitor combined with anti‐angiogenesis group, the pooled 3‐, 6‐, 12‐, and 18‐month PFS rates were 83.9% [95% CI: 0.747–0.914, *I* ^2^ = 0%], 56.3% [95% CI: 0.451–0.671, *I* ^2^ = 0%], 34.8% [95% CI: 0.246–0.458, *I* ^2^ = 12%], and 8.6% [95% CI: 0–0.525, *I* ^2^ = 95%], respectively (Figure S10A–D). The pooled 3‐, 6‐, 12‐, and 18‐month OS rates were 90.2% [95% CI: 0.832–0.957, *I* ^2^ = 0%], 80.3% [95% CI: 0.716–0.878, *I* ^2^ = 0%], 54.1% [95% CI: 0.440–0.640, *I* ^2^ = 50%], and 19.7% [95% CI: 0–0.668, *I* ^2^ = 95%], respectively (Figure S10E–H). In the PD‐1 inhibitor combined with chemotherapy group, the pooled 3‐, 6‐, 12‐, and 18‐month PFS rates were 85.0% [95% CI: 0.792–0.901, *I* ^2^ = 0%], 62.9% [95% CI: 0.554–0.700, *I* ^2^ = 0%], 33.7% [95% CI: 0.267–0.410, *I* ^2^ = 0%], and 21.9% [95% CI: 0.160–0.285, *I* ^2^ = 0%], respectively (Figure S11A–D). The pooled 3‐, 6‐, 12‐, and 18‐month OS rates were 96.0% [95% CI: 0.924–0.986, *I* ^2^ = 0%], 87.3% [95% CI: 0.818–0.920, *I* ^2^ = 0%], 59.4% [95% CI: 0.515–0.667, *I* ^2^ = 48%], and 47.7% [95% CI: 0.402–0.552, *I* ^2^ = 21%], respectively (Figure S11E–H). The pooled 3‐, 6‐, and 12‐month PFS and OS rates were similar between the two groups, while the 18‐month PFS and OS rates were significantly higher in the PD‐1 inhibitor combined with chemotherapy group than in the PD‐1 inhibitor combined with anti‐angiogenesis group (Figure 5E,F).

### 3.7 Analysis of TRAEs

Pooled incidence rates of TRAEs are shown in Table 1. The most commonly observed TRAEs were grade 1–2, including hematologic toxicity, gastrointestinal reactions, skin reactions, anti‐angiogenesis‐related adverse events, and immunotherapy‐related adverse events. The pooled incidence rates of most TRAEs (any grade) in the combination immunotherapy group were similar to those in the chemotherapy group, except for hypothyroidism, pneumonia, lymphopenia, fatigue, and anorexia. Moreover, the pooled incidence of grade ≥ 3 hematologic toxicities was substantially lower in the combination immunotherapy group than in chemotherapy, other than lymphopenia. By contrast, when compared with the PD‐1 inhibitor monotherapy group, most TRAEs occurred more frequently in the combination immunotherapy group.

**TABLE 1:** Pooled incidence rates of adverse events among the PD‐1 inhibitor monotherapy, chemotherapy, and combination immunotherapy groups.

		PD‐1 inhibitors monotherapy	Chemotherapy	Combination immunotherapy	*p* 1	*p* 2
All events	Any grade	78.6%	94.0%	89.4%	0.04	< 0.01
	Grade ≥ 3	26.4%	56.1%	41.5%	< 0.01	< 0.01
Anemia	Any grade	8.8%	30.8%	30.9%	0.96	< 0.01
	Grade ≥ 3	1.3%	7.0%	1.2%	< 0.01	0.71
Leukopenia	Any grade	8.1%	37.0%	37.7%	0.82	< 0.01
	Grade ≥ 3	—	21.1%	7.9%	< 0.01	—
Neutropenia	Any grade	2.4%	28.7%	23.3%	0.08	< 0.01
	Grade ≥ 3	—	23.0%	4.7%	< 0.01	—
Thrombocytopenia	Any grade	—	—	23.0%	—	—
	Grade ≥ 3	—	—	2.2%	—	—
Lymphopenia	Any grade	12.4%	4.8%	41.0%	< 0.01	< 0.01
	Grade ≥ 3	0.8%	1.2%	12.2%	< 0.01	< 0.01
Nausea	Any grade	2.3%	26.7%	—	—	—
	Grade ≥ 3	—	1.3%	—	—	—
Vomiting	Any grade	1.5%	22.2%	—	—	—
	Grade ≥ 3	—	4.5%	—	—	—
Diarrhea	Any grade	6.6%	20.7%	16.2%	0.08	< 0.01
	Grade ≥ 3	0.3%	2.5%	1.4%	0.24	0.19
Fatigue	Any grade	8.1%	22.5%	32.2%	0.01	< 0.01
	Grade ≥ 3	0.9%	2.6%	2.0%	0.70	0.21
Anorexia	Any grade	6.1%	27.1%	39.2%	0.01	< 0.01
	Grade ≥ 3	0.5%	3.9%	—	—	—
Pneumonia	Any grade	10.1%	3.4%	17.5%	< 0.01	0.05
	Grade ≥ 3	3.5%	—	—	—	—
RCCEP	Any grade	—	—	16.1%	—	—
	Grade ≥ 3	—	—	1.6%	—	—
Hypertension	Any grade	—	—	27.2%	—	—
	Grade ≥ 3	—	—	3.3%	—	—
Proteinuria	Any grade	—	—	14.4%	—	—
Rash	Any grade	11.6%	9.3%	11.4%	0.45	0.99
Hypothyroidism	Any grade	12.9%	0.5%	19.6%	< 0.01	< 0.01
Alopecias	Any grade	—	30.0%	—	—	—

*Note:* *p*‐value 1, represent results of chi‐square tests between the chemotherapy group and the combination immunotherapy group. *p*‐value 2, represent results of chi‐square tests between the PD‐1 inhibitors monotherapy group and the combination immunotherapy group. — indicates data not available.

Abbreviation: RCCEP, reactive cutaneous capillary endothelial proliferation.

## 4 Discussion

In this study, we conducted a pooled analysis of current data to evaluate the clinical efficacy of second‐ or later‐line therapies for ESCC patients and to explore the potential of PD‐1 inhibitor‐based combination therapies compared with previous standard treatments. Our findings demonstrated that PD‐1 inhibitor‐based combination therapies offer significantly higher ORR, DCR, and improved outcomes compared to PD‐1 inhibitor monotherapy or chemotherapy. Notably, second‐line PD‐1 inhibitor‐based combination therapies showed more clinical benefits as compared with the standard PD‐1 inhibitor monotherapy in immunotherapy‐naive ESCC patients. Importantly, PD‐1 inhibitor combined with anti‐angiogenesis therapy or chemotherapy also showed high antitumor activity in previously immunotherapy‐treated advanced ESCC patients, indicating strong potential for future treatment strategies, providing a promising second‐line treatment option as current standards face challenges. PD‐1 inhibitor‐based combination therapy was associated with higher treatment‐related but manageable toxicity compared with PD‐1 inhibitor monotherapy.

Currently, clinicians face a dilemma regarding the standard second‐line treatment of PD‐1 inhibitor monotherapy for advanced ESCC, as patients with a good performance status achieve a mOS of only 7.2–10.9 months [18, 20, 22, 30, 31]. Furthermore, there are no optimal treatment recommendations for ESCC patients who progress after first‐line immunotherapy. In patients with immunotherapy‐naive advanced ESCC, the ATTRACTION‐3 trial has demonstrated that nivolumab significantly improved OS compared to chemotherapy (mOS: 10.9 vs. 8.5 months, *p* = 0.0264), with 3‐year OS rates of 15.3% in the nivolumab group versus 8.7% in the chemotherapy group [21]. Notably, 118 patients (56.2%) in the nivolumab group received subsequent systemic therapy, including fluoropyrimidine‐based chemotherapy (29 patients), platinum‐based chemotherapy (25 patients), and taxane therapies (106 patients). Similarly, 104 patients (49.8%) in the chemotherapy group received subsequent systemic therapy, including fluoropyrimidine‐based chemotherapy (40 patients), platinum‐based chemotherapy (22 patients), and taxane therapies (45 patients) [21]. Therefore, a substantial proportion of patients with ESCC can benefit from second‐line PD‐1 inhibitor monotherapy and also demonstrate good tolerance to the subsequent chemotherapy, suggesting the possibility of optimizing PD‐1 inhibitor‐based second‐line treatment strategies to further enhance the efficacy beyond PD‐1 inhibitor monotherapy alone.

To date, with the increasing number of patients being treated with ICIs combination therapy, single‐agent immunotherapy is no longer sufficient to meet clinical needs and patients' demands. Several reviews have extensively discussed combination strategies aimed at maximizing the benefits of cancer immunotherapy [32, 33]. These combinations include PD‐1 inhibitor combined with chemotherapy, radiotherapy, anti‐angiogenesis therapy, small‐molecule tumor‐targeted therapy, third‐generation immune checkpoint inhibitors (e.g., LAG3, TIGIT, and TIM3), as well as other approaches (such as oncolytic viruses, strategies to stimulate innate immune cells, cytokine‐based therapy, adoptive cell therapy, and T cell engagers) [32, 33]. Considering the accessibility, convenience, and operability of combination treatment strategies in clinical significance, immunotherapy combined with chemotherapy, radiotherapy, and anti‐angiogenesis therapy currently stands as a more effective option. Mechanistically, chemotherapy can promote anticancer immune responses by inducing immunogenic cell death (ICD), activating immune effector cells, and impairing the functions of immunosuppressive cells, thus enhancing the efficacy of immunotherapy [34, 35]. Radiotherapy similarly induces anticancer immunity by promoting ICD, upregulating MHC class I expression, enhancing tumor antigen presentation, downregulating CD47 mediated signals, and producing reactive oxygen species [32, 33]. Since aberrant angiogenesis in tumors is linked to immune evasion and immunosuppression [36, 37, 38], angiogenesis inhibitors have been shown to favorably modulate the tumor microenvironment (TME) by increasing the infiltration of neutrophils and mature dendritic cells while reducing myeloid‐derived suppressor cells (MDSCs), regulatory T cells (Tregs), and macrophages [39, 40]. Clinically, these combination strategies did show promise in improving the efficacy of cancer immune treatments. PD‐1/PD‐L1 inhibitors combined with chemotherapy have been extensively approved by the FDA for various cancer types, including ESCC. Similarly, PD‐1/PD‐L1 inhibitor combined with anti‐angiogenesis (such as axitinib, cabozantinib, lenvatinib, and bevacizumab) has been FDA‐approved for kidney cancer, endometrial cancer, and hepatocellular carcinoma [33]. Clinical data also suggest synergy between immunotherapy and radiotherapy, although results remain limited and sometimes controversial across different cancers [41, 42]. In our study, we found that PD‐1 inhibitor combined with various treatments including chemotherapy, anti‐EGFR, anti‐angiogenesis (surufatinib, anlotinib, and apatinib), anti‐TIGIT therapies, and radiotherapy demonstrated synergistic efficacy in the second‐ or later‐line treatment of advanced ESCC. These combinations led to higher ORR, DCR, and improved survival compared to standard second‐line immunotherapy monotherapy or chemotherapy. Since different tyrosine kinase inhibitors (TKIs) like surufatinib, anlotinib, apatinib, axitinib, cabozantinib, lenvatinib, and sitravatinib share similar anti‐angiogenesis effects by targeting VEGFR family [43, 44, 45, 46], and trials such as LEAP‐004 (NCT03776136) and LEAP‐005 (NCT03797326) are investigating the combination of lenvatinib and pembrolizumab for second‐ or later‐line treatment in solid tumors [47], these combination strategies are becoming increasingly accepted in clinical practice in both Eastern and Western settings. Although many of these combination therapies originated in China, the data suggest that combining immunotherapy with commonly used anti‐angiogenesis agents or chemotherapy offers a feasible and effective approach to improve second‐line treatment efficacy for advanced ESCC.

Currently, there are no recommendations for second‐ or later‐line treatment options for immunotherapy‐treated ESCC across all treatment guidelines. It is estimated that around 61% of NSCLC patients eventually develop acquired resistance to ICIs [48]. Therefore, in the near future, the situation for a large population of ESCC patients suffering from acquired resistance to immunotherapy may become increasingly severe, as first‐line immunotherapy for advanced ESCC offers only 5.7–7.3 months of mPFS. The mechanisms of acquired resistance to ICIs are multifactorial, complex, and dynamic, involving impairments in antigen presentation machinery, defects in IFN‐γ signaling, neoantigen depletion, tumor‐mediated immunosuppression or exclusion, upregulation of alternative T‐cell checkpoints (e.g., TIM3, LAG3, V‐domain immunoglobulin suppressor of T‐cell activation), and changes in the tumor microenvironment (TME) [23, 49, 50, 51]. Currently, strategies to address acquired resistance to ICIs include chemotherapy, radiotherapy, targeted therapies, other immunotherapeutic agents, rechallenging with immunotherapy beyond progression, or combinations of these approaches [43, 50]. Yamamoto et al. reported that for small cell lung cancer treated with continuous immunotherapy beyond progression, the mPFS, time to treatment failure (TTF), and OS were 6.7 months, 21.4 months, and 39.3 months, respectively [52]. However, both LEAP‐008 (lenvatinib plus pembrolizumab, NCT03976375) and SAPPHIRE trial (sitravatinib plus nivolumab, NCT03906071) for NSCLC progressed after ICIs did not improve patients' efficacy as compared with docetaxel [53, 54]. In ESCC, the CAP 02 Rechallenge trial demonstrated that patients treated with camrelizumab plus apatinib after progression on immune checkpoint inhibitors had an ORR of 36.8%, DCR of 89.5%, and a median duration of response (mDOR), median time to response (mTOR), mPFS, and mOS of 3.0 months, 2.2 months, 4.6 months, and 7.5 months, respectively [27]. Recently, a multicenter, retrospective, real‐world clinical study showed that mPFS and mOS were 5.6 and 11.1 months in previously ICI‐treated advanced ESCC patients who received anlotinib plus PD‐1 inhibitors treatment [55]. Due to the limited data on this patient population during the study search period, we here performed the updated pooled analysis upon the publish of ChiCTR2300070777 study to assess the treatment efficacy of combined immunotherapy in ESCC patients who develop acquired resistance to ICIs. The pooled ORR, DCR, 3‐, 6‐, 12‐, and 18‐month PFS, 3‐, 6‐, 12‐, 18‐, and 24‐month OS rates were 32.5% [95% CI: 0.164–0.509, *I* ^2^ = 83%], 81.1% [95% CI: 0.660–0.926, *I* ^2^ = 80%], 76.1% [95% CI: 0.700–0.817, *I* ^2^ = 0%], 45.3% [95% CI: 0.342–0.566, *I* ^2^ = 60%], 21.8% [95% CI: 0.164–0.278, *I* ^2^ = 46%], 10.1% [95% CI: 0.002–0.295, *I* ^2^ = 91%], 91.1% [95% CI: 0.802–0.982, *I* ^2^ = 71%], 80.5% [95% CI: 0.653–0.922, *I* ^2^ = 76%], 48.4% [95% CI: 0.406–0.563, *I* ^2^ = 0%], and 35.8% [95% CI: 0.284–0.435, *I* ^2^ = 0%] respectively (Figure S12A–J). These findings offer valuable insights for clinicians seeking to optimize treatment strategies for managing acquired resistance to ICIs. Notably, future strategies that focus on combining ICIs with anti‐angiogenesis agents or chemotherapy might show promising potential and are highly anticipated.

These findings might have important implications for clinical practice, particularly in regions where access to newer therapies may be limited, such as low‐ and middle‐income countries. The demonstrated efficacy of PD‐1 inhibitor‐based combination therapies as second‐ or later‐line treatment suggests that patients who have progressed on prior lines of therapy may achieve meaningful clinical benefit when combination strategies are feasible. In settings with restricted access to novel agents, clinicians may consider prioritizing combinations that utilize widely available therapies, such as chemotherapy or radiotherapy, alongside PD‐1 inhibitors, to optimize treatment outcomes. Our findings also support a pragmatic selection of second‐ or later‐line treatment (combining ICIs with either anti‐angiogenesis agents or chemotherapy) to balance clinical efficacy, safety, and resource availability because PD‐1 inhibitor combined with anti‐angiogenesis therapy or chemotherapy showed high antitumor activity in both immunotherapy‐naive and immunotherapy‐treated advanced ESCC patients.

This study has some limitations. First, the included studies on PD‐1 inhibitor‐based combination therapies were single‐arm or cohort studies with relatively small sample sizes. This contributed to substantial heterogeneity in the pooled estimates and may have introduced selection bias, thereby weakening the strength of the evidence. Well‐powered randomized controlled trials are needed to further validate these results and to evaluate key clinical outcomes. Second, combined immunotherapy strategies—including chemotherapy, radiotherapy, and targeted therapy—are already widely applied in clinical oncology. However, the diversity of regimens and the use of different PD‐1 inhibitors in the included studies introduced additional heterogeneity, which limited our ability to determine the most effective combinations. Third, all participants in the studies evaluating immunotherapy combined with anti‐angiogenic agents were from China, which may have introduced population selection bias and limited the generalizability of the findings.

In conclusion, this systematic review and meta‐analysis demonstrated that combination immunotherapy offers additional clinical benefits compared to standard immunotherapy monotherapy in the second‐line treatment of ESCC, as evidenced by improved ORR, DCR, and survival rates. Anti‐angiogenesis drugs or chemotherapy hold significant potential for clinical practice in both immunotherapy‐naive and immunotherapy‐refractory patients. However, high‐level evidence remains lacking, and further large‐scale randomized controlled trials are warranted to clarify the potential benefits of these combination strategies.

## Author Contributions

Conception and design: Yong Chen and Ying Li. Collection and assembly of data: Zixuan Chen and Hong Guo. Data analysis and interpretation: Rui Wang, Fang Han, Haiyan Mao, and Zhengrong Zhang. Manuscript writing: All authors. Final approval of manuscript: All authors. Accountable for all aspects of the work: All authors.

## Conflicts of Interest

The authors declare no conflicts of interest.

## Supporting information


**Appendix S1:** cam471329‐sup‐0001‐AppendixS1.docx.

## Data Availability

The data that support the findings of this study are available from the corresponding author upon reasonable request.
