# Supplementary material for: Clinical Benefits of Combination Immunotherapy Over Standard Immunotherapy Monotherapy in Previously Treated Advanced Esophageal Squamous Cell Carcinoma: A Systematic Review and Meta‐Analysis
Source: Cancer Med. 2025 Oct 29;14(21):e71329. doi: 10.1002/cam4.71329 (PMC12572628; doi:10.1002/cam4.71329)
Supplement: Supplementary file 1 — Appendix S1: cam471329‐sup‐0001‐AppendixS1.docx. [file CAM4-14-e71329-s001.docx]

| Supplementary Table 1 Seach strategies in different database |
| --- |
| Embase and Medline |
| 1 (esophageal OR esophagus OR oesophageal OR oesophagus OR gastroesophageal OR oesophagogastric OR esophagogastric) AND (cancer OR cancers OR tumor OR tumour OR tumors OR tumours OR neoplasm OR neoplasms OR malignancy OR malignancies OR adenocarcinoma OR adenocarcinomas OR carcinoma OR carcinomas) |
| 2 esophagus tumor |
| 3 1 OR 2 |
| 4 immunotherapy OR PD-1 checkpoints inhibitors OR PD-1 blockade OR immune checkpoint inhibitor OR ICI OR immune checkpoint blocking agent OR immune checkpoint blockade OR immunotherapy OR immunotherapies OR immunosuppression OR nivolumab OR opdivo OR pembrolizumab OR keytruda OR atezolizumab OR tecentriq OR durvalumab OR imfinzi OR camrelizumab OR sintilimab OR tislelizumab OR toripalimab OR avelumab OR ipilimumab OR tremelimumab OR bavencio OR lambrolizumab OR pidilizumab OR cemiplimab OR envafolimab OR bintrafusp OR cetrelimab OR Ticilimumab OR Opdivo OR BGBA317 OR BGB-A317 OR Yervoy OR programmed cell death1 OR PD-1 OR PD1 OR PD 1 OR anti-PD-1 OR programmed cell death-Ligand1 OR PD-L1 OR PDL1 OR PDL1 OR anti-PD-L1 OR Cytotoxic T lymphocyte antigen-4 OR CTLA-4 OR CTLA4 OR anti-CTLA-4 |
| 5 second-line OR second line or second-line procedure OR second-line therapy OR second-line treatment OR second line procedure OR second line therapy OR second line treatment OR third-line OR third line or third-line procedure OR third-line therapy OR third-line treatment OR third line procedure OR third line therapy OR third line treatmentpretreat OR previously treated OR refracrory OR recurrent |
| 6 3 AND 4 AND 5 |

| Web of Science |
| --- |
| 1 TS=(esophageal OR esophagus OR oesophageal OR oesophagus OR gastroesophageal OR oesophagogastric OR esophagogastric) AND (cancer OR cancers OR tumor OR tumour OR tumors OR tumours OR neoplasm OR neoplasms OR malignancy OR malignancies OR adenocarcinoma OR adenocarcinomas OR carcinoma OR carcinomas) |
| 2 TS=esophagus tumor |
| 3 1 OR 2 |
| 4 TS=(immunotherapy OR PD-1 checkpoints inhibitors OR PD-1 blockade OR immune checkpoint inhibitor OR ICI OR immune checkpoint blocking agent OR immune checkpoint blockade OR immunotherapy OR immunotherapies OR immunosuppression OR nivolumab OR opdivo OR pembrolizumab OR keytruda OR atezolizumab OR tecentriq OR durvalumab OR imfinzi OR camrelizumab OR sintilimab OR tislelizumab OR toripalimab OR avelumab OR ipilimumab OR tremelimumab OR bavencio OR lambrolizumab OR pidilizumab OR cemiplimab OR envafolimab OR bintrafusp OR cetrelimab OR Ticilimumab OR Opdivo OR BGBA317 OR BGB-A317 OR Yervoy OR programmed cell death1 OR PD-1 OR PD1 OR PD 1 OR anti-PD-1 OR programmed cell death-Ligand1 OR PD-L1 OR PDL1 OR PDL1 OR anti-PD-L1 OR Cytotoxic T lymphocyte antigen-4 OR CTLA-4 OR CTLA4 OR anti-CTLA-4) |
| 5 TS=(second-line OR second line or second-line procedure OR second-line therapy OR second-line treatment OR second line procedure OR second line therapy OR second line treatment OR third-line OR third line or third-line procedure OR third-line therapy OR third-line treatment OR third line procedure OR third line therapy OR third line treatmentpretreat OR previously treated OR refracrory OR recurrent) |
| 6 3 AND 4 AND 5 |

| Cochrane Collaboration database |
| --- |
| 1 (esophageal OR esophagus OR oesophageal OR oesophagus OR gastroesophageal OR oesophagogastric OR esophagogastric) AND (cancer OR cancers OR tumor OR tumour OR tumors OR tumours OR neoplasm OR neoplasms OR malignancy OR malignancies OR adenocarcinoma OR adenocarcinomas OR carcinoma OR carcinomas):ti,ab,kw |
| 2 MeSH descriptor: [Esophageal Neoplasms] explode all trees |
| 3 1 OR 2 |
| 4 (immunotherapy OR PD-1 checkpoints inhibitors OR PD-1 blockade OR immune checkpoint inhibitor OR ICI OR immune checkpoint blocking agent OR immune checkpoint blockade OR immunotherapy OR immunotherapies OR immunosuppression OR nivolumab OR opdivo OR pembrolizumab OR keytruda OR atezolizumab OR tecentriq OR durvalumab OR imfinzi OR camrelizumab OR sintilimab OR tislelizumab OR toripalimab OR avelumab OR ipilimumab OR tremelimumab OR bavencio OR lambrolizumab OR pidilizumab OR cemiplimab OR envafolimab OR bintrafusp OR cetrelimab OR Ticilimumab OR Opdivo OR BGBA317 OR BGB-A317 OR Yervoy OR programmed cell death1 OR PD-1 OR PD1 OR PD 1 OR anti-PD-1 OR programmed cell death-Ligand1 OR PD-L1 OR PDL1 OR PDL1 OR anti-PD-L1 OR Cytotoxic T lymphocyte antigen-4 OR CTLA-4 OR CTLA4 OR anti-CTLA-4):ti,ab,kw |
| 5 (second-line OR second line or second-line procedure OR second-line therapy OR second-line treatment OR second line procedure OR second line therapy OR second line treatment OR third-line OR third line or third-line procedure OR third-line therapy OR third-line treatment OR third line procedure OR third line therapy OR third line treatmentpretreat OR previously treated OR refracrory OR recurrent):ti,ab,kw |
| 6 3 AND 4 AND 5 |

| Supplementary Table 2 Characteristics of included studies | | | | | | | | |
| --- | --- | --- | --- | --- | --- | --- | --- | --- |
| Study | Year | Country | Treatment line | n | Treatment arms | median follow-up  (months) | mPFS | mOS |
|  |  |  |  |  |  |  |  |  |
| Ohsawa M^[1]^ | 2023 | China | 2L+3L | 61 | Nivolumab | NA | 4.2 | 8.4 |
|  |  |  | 2L+3L | 105 | Paclitaxel | NA | 2.8 | 8.2 |
| Yang GL^[2]^ | 2022 | China | 2L | 18 | Immunotherapy | 13.9 | 3.2 | 9.8 |
|  |  |  | 2L | 151 | Combination immunotherapy | 14.0 | 8.5 | 18.9 |
| Kim JH^[3]^ | 2022 | Korea | 2L+3L | 60 | Nivolumab/Pembrolizumab | 16.0 | 1.9 | 6.4 |
| Li XY^[4]^ | 2022 | China | 2L | 42 | Chemotherapy | 13.5 | 7.1 | NA |
|  |  |  | 2L | 106 | PD-1 inhibitors | 14.1 | 7.4 | 14.1 |
| Xu JM^[5]^ | 2022 | China | 2L | 95 | Sintilimab | 7.2 | 1.6 | 7.2 |
|  |  |  | 2L | 95 | Paclitaxel/Irinotecan | 6.2 | 2.9 | 6.2 |
| Lu M^[6]^ | 2021 | China | 2L | 20 | Surufatinib+Toripalimab | 9.72 | 2.7 | 10.4 |
| Wang F^[7]^ | 2023 | Global | 2L | 62 | Ociperlimab+Tislelizumab | NA | 3.6 | 10.1 |
|  |  |  | 2L | 63 | Tislelizumab | NA | 2.8 | 9.3 |
| Huang J^[8]^ | 2020 | China | 2L | 228 | Camrelizumab | 8.3 | 1.9 | 8.3 |
|  |  |  | 2L | 220 | Paclitaxel/Irinotecan | 6.2 | 1.9 | 6.2 |
| Kojima T^[9]^ | 2020 | Global | 2L | 198 | Pembrolizumab | 7.1 | 2.2 | 8.2 |
|  |  |  | 2L | 203 | Paclitaxel/Irinotecan | 6.9 | 3.1 | 7.1 |
| Huang J^[10]^ | 2023 | China | 2L+3L | 96 | Anlotinib+PD-1 inhibitors | NA | 6.3 | 11.0 |
| Liu Y^[11]^ | 2022 | China | 2L+3L | 46 | Anlotinib+PD-1 inhibitors | 9.3 | 5.4 | NA |
| Shen L^[12]^ | 2022 | Global | 2L | 256 | Tislelizumab | 8.5 | 1.6 | 8.6 |
|  |  |  | 2L | 256 | Paclitaxel/Docetaxel/Irinotecan | 5.8 | 2.1 | 6.3 |
| Kato K^[13]^ | 2022 | Global | 2L | 210 | Nivolumab | 10.5 | 1.7 | 10.9 |
|  |  |  | 2L | 209 | Chemotherapy | 8.0 | 3.4 | 8.4 |
| Xia J^[14]^ | 2023 | China | 2L | 28 | Camrelizumab+Nimotuzumab | NA | NA | NA |
| Meng XR^[15]^ | 2022 | China | 2L | 52 | Camrelizumab+Apatinib | 7.5 | 6.8 | 15.8 |
| Meng XR^[16]^* | 2023 | China | 2L | 49 | Camrelizumab+Apatinib | 5.2 | 4.6 | 7.5 |
| He YF^[17]^ | 2022 | China | 2L | 16 | Camrelizumab+Apatinib+Irinotecan | NA | NA | NA |
| Saori M^[18]^ | 2023 | Japan | 2L+3L | 13 | Nivolumab | NA | 3.5 | 12.7 |
| Zhao WS^[19]^ | 2023 | China | 2L | 49 | Camrelizumab+Irinotecan+Radiationtherapy | 12.8 | 6.9 | 12.8 |
| Abbreviations: 2L, second-line therapy; 3L, third-line therapy; PD-1, programmed cell death protein 1; mPFS, median progression-free survival; mOS, median overall survival; NA, data was not available; *, CAP 02 rechallenge study. | | | | | | | | |

1. Ohsawa M, Hamai Y, Emi M, et al. Real-world clinical outcomes of nivolumab and taxane as a second- or later-line therapy for recurrent or unresectable advanced esophageal squamous cell carcinoma [J]. Frontiers in Oncology, 2023, 13: 1126536.

2. Yang G, Sun H, Zhou C, et al. PD-1 inhibitor monotherapy versus combination therapy [J]. Journal of Cancer Research and Therapeutics, 2022, 18(2): 545-52.

3. Kim J H, Ahn B, Hong S-M, et al. Real-World Efficacy Data and Predictive Clinical Parameters for Treatment Outcomes in Advanced Esophageal Squamous Cell Carcinoma Treated with Immune Checkpoint Inhibitors [J]. Cancer Research and Treatment, 2022, 54(2): 505-16.

4. Li XY, Huang LS, Cai HQ, et al. First‐line or second‐line PD‐1 inhibition in advanced oesophageal squamous cell carcinoma: A prospective, multicentre, registry study [J]. Journal of Clinical Pharmacy and Therapeutics, 2022, 47(6): 732-7.

5. Xu J, Li Y, Fan Q, et al. Clinical and biomarker analyses of sintilimab versus chemotherapy as second-line therapy for advanced or metastatic esophageal squamous cell carcinoma: a randomized, open-label phase 2 study (ORIENT-2) [J]. Nature Communications, 2022, 13(1): 857.

6. Zhang P, Chen Z, Shi S, et al. Efficacy and safety of surufatinib plus toripalimab, a chemotherapy-free regimen, in patients with advanced gastric/gastroesophageal junction adenocarcinoma, esophageal squamous cell carcinoma, or biliary tract cancer [J]. Cancer Immunology, Immunotherapy, 2024, 73(7):119.

7. Wang F, Lin C Y, Sun J M, et al. AdvanTIG-203: Phase II randomized, multicenter study of ociperlimab (OCI) + tislelizumab (TIS) in patients (pts) with unresectable, locally advanced, recurrent/metastatic esophageal squamous cell carcinoma (ESCC) and programmed cell death-ligand 1 (PD-L1) positivity [J]. Annals of Oncology, 2023, 34: S621.

8. Huang J, Xu J, Chen Y, et al. Camrelizumab versus investigator's choice of chemotherapy as second-line therapy for advanced or metastatic oesophageal squamous cell carcinoma (ESCORT): a multicentre, randomised, open-label, phase 3 study [J]. The Lancet Oncology, 2020, 21(6): 832-42.

9. Kojima T, Shah MA , Muro K, et al. Randomized Phase III KEYNOTE-181 Study of Pembrolizumab Versus Chemotherapy in Advanced Esophageal Cancer [J]. Journal of Clinical Oncology, 2020, 38(35): 4138-48.

10. Huang J, Liu J, Hong Y, et al. Preliminary results of the feasibility and tolerability of anlotinib plus PD-1 blockades among patients with previously immunotherapy treated advanced esophageal squamous cell carcinoma (ESCC): A retrospective exploratory study [J]. Annals of Oncology, 2023, 34: S73.

11. Liu Y, Ge Q, Xu S, et al. Efficacy and safety of anlotinib plus programmed death-1 blockade versus anlotinib monotherapy as second or further-line treatment in advanced esophageal squamous cell carcinoma: A retrospective study [J]. Frontiers in Oncology, 2022, 12: 942678.

12. Shen L, Kato K , Kim SB, et al. Tislelizumab Versus Chemotherapy as Second-Line Treatment for Advanced or Metastatic Esophageal Squamous Cell Carcinoma (RATIONALE-302) A Randomized Phase III Study [J]. Journal of Clinical Oncology. 2022, 40(26): 3065-76.

13. Kato K, Cho BC, Takahashi M, et al. Nivolumab versus chemotherapy in patients with advanced oesophageal squamous cell carcinoma refractory or intolerant to previous chemotherapy (ATTRACTION-3): a multicentre, randomised, open-label, phase 3 trial [J]. The Lancet Oncology, 2019, 20(11): 1506-17.

14. Xia J, Meng XR, Ji YH, et al. Second-Line Of Camrelizumab Plus Nimotuzumab In Advanced Esophageal Squamous Cell Carcinoma (ESCC): A Single-Arm, Open-Label, Phase 2 Trial [J]. Diseases of the Esophagus, 2023, 36.

15. Meng XR, Wu T, Hong Y, et al. Camrelizumab plus apatinib as second-line treatment for advanced oesophageal squamous cell carcinoma (CAP 02): a single-arm, open-label, phase 2 trial [J]. The Lancet Gastroenterology & Hepatology, 2022, 7(3): 245-53.

16. Meng Xr, Wang J, Xia J, et al. Efficacy and safety of camrelizumab plus apatinib in patients with advanced esophageal squamous cell carcinoma previously treated with immune checkpoint inhibitors (CAP 02 Re-challenge): A single-arm, phase II study [J]. European Journal of Cancer, 2024, 212: 114328.

17. He Y, Li C, Zhang F, et al. Clinical study on the second-line treatment of advanced esophageal squamous cell carcinoma with camrelizumab combined with apatinib and irinotecan: A single-arm, multicenter, phase II study [J]. Journal of Clinical Oncology, 2022, 40: 319.

18. Miyajima S, Tsuji K, Kito Y. Nivolumab monotherapy in second- or later-line treatment for advanced or recurrent esophageal cancer [J]. Annals of Oncology, 2023, 34: S1436.

19. Zhao WS, Ke S, Cai X, et al. Radiotherapy plus camrelizumab and irinotecan for oligometastatic esophageal squamous cell carcinoma patients after first-line immunotherapy plus chemotherapy failure: An open-label, single-arm, phase II trial [J]. Radiotherapy and Oncology, 2023, 184: 109679.

| Supplementary Table 3. Quality assessment (Cochrane reviews) of Randomized controlled trials | | | | | | | |
| --- | --- | --- | --- | --- | --- | --- | --- |
| study | Random sequence generation | Allocation concealment | Blinding of participants and personnel | Blinding of outcome assessment | Incomplete outcome data | Selective reporting | Other bias |
| Shen L 2022 | + | ? | - | ? | + | + | + |
| Xu JM 2022 | + | + | ? | ? | + | + | + |
| Kojima T 2020 | + | + | ? | + | + | + | + |
| Kato K 2019 | + | + | - | + | + | + | + |
| Huang J 2020 | + | + | + | + | + | + | + |
| Wang F 2023 | ? | ? | + | + | + | + | + |

| Supplementary Table 4. Quality assessment (NOS) of cohort studies | | | | | | | | | | |
| --- | --- | --- | --- | --- | --- | --- | --- | --- | --- | --- |
| Study | Cohort selection | | | |  | Comparability |  | Outcome ascertainment | | |
|  | A | B | C | D |  | E |  | F | G | H |
| Ohsawa M 2023 | 1 | 1 | 1 | 1 |  | 1 |  | 1 | 1 | 1 |
| Yang GL 2022 | 1 | 1 | 1 | 1 |  | 1 |  | 1 | 0 | 1 |
| Li XY 2022 | 1 | 1 | 1 | 1 |  | 1 |  | 1 | 1 | 1 |
| Liu Y 2022 | 1 | 1 | 1 | 1 |  | 1 |  | 0 | 0 | 1 |
| A:Representativeness of the Exposed Cohort  B:Selection of the Non-Exposed Cohort  C:Ascertain-ment of Exposure  D:Demonstration that Outcome of Interest Was Not Present at Start of Study  E:Comparability of Cases and Controls on the Basis of the Design or Analysis  F:Assessment of Outcome  G:Was Follow-Up Long Enough for Outcomes to Occur  H:Adequacy of Follow Up of Cohorts | | | | | | | | | | |

| Supplementary Table 5. Quality assessment (JBI) of Single-arm studies | | | | | | | | | | |
| --- | --- | --- | --- | --- | --- | --- | --- | --- | --- | --- |
| study | 1 | 2 | 3 | 4 | 5 | 6 | 7 | 8 | 9 | 10 |
| Kim JH 2022 | + | + | + | + | + | + | + | + | + | + |
| Lu M 2021 | + | + | + | - | - | + | - | + | - | + |
| Huang J 2023 | + | + | + | + | + | + | - | + | - | + |
| Xia J 2023 | + | + | + | + | + | + | + | + | ？ | + |
| Meng XR 2022 | + | + | + | + | + | + | + | + | + | + |
| Meng XR 2023*  (rechallenge) | + | + | + | + | - | + | - | - | - | + |
| He YF 2022 | + | + | + | - | - | + | - | + | - | + |
| Saori M 2023 | + | + | + | + | + | + | + | + | + | + |
| Zhao WS 2023 | + | + | + | + | + | + | + | + | + | + |
| 1: Were there clear criteria for inclusion in the case series?  2: Was the condition measured in a standard, reliable way for all participants included in the case series?  3: Were valid methods used for identification of the condition for all participants included in the case series?  4: Did the case series have consecutive inclusion of participants?  5: Did the case series have complete inclusion of participants?  6: Was there clear reporting of the demographics of the participants in the study?  7: Was there clear reporting of clinical information of the participants?  8: Were the outcomes or follow-up results of cases clearly reported?  9: Was there clear reporting of the presenting site(s)/clinic(s) demographic information?  10: Was statistical analysis appropriate? | | | | | | | | | | |

**
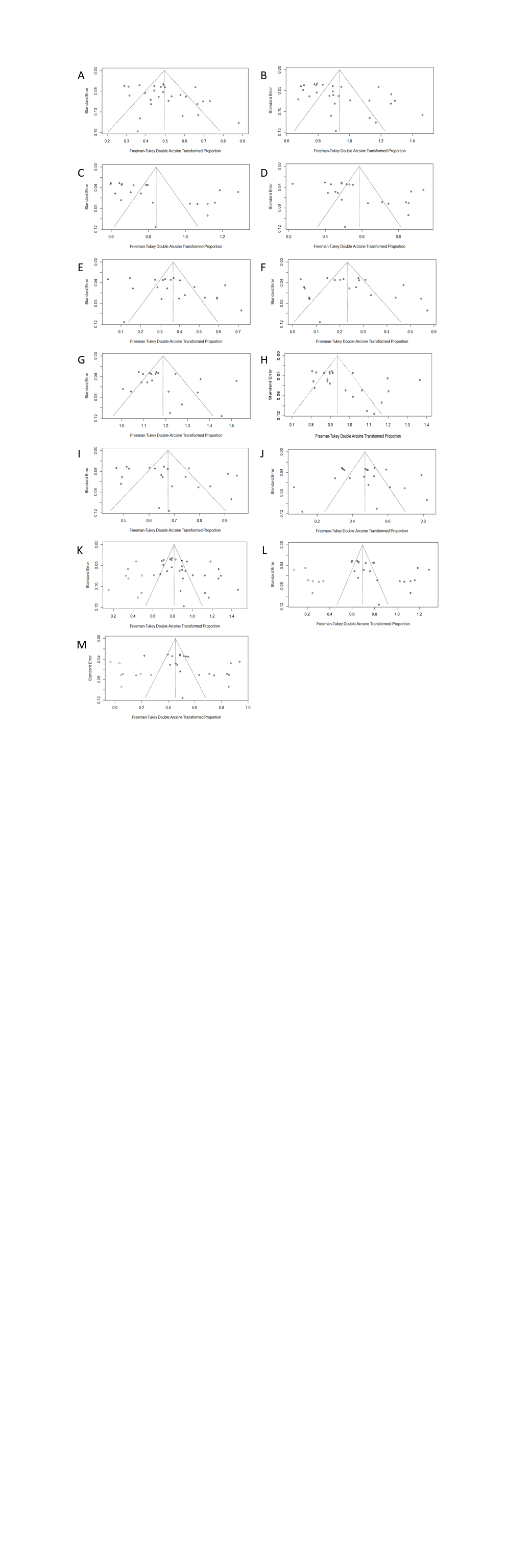
Supplementary Figure 1.** Funnel plots for publication bias of ORR (A), DCR (B), 3-, 6-, 12- and 18-month PFS rates (C-F), 3-, 6-, 12- and 18-month OS rates (G-J). Adjusted Funnel plots for DCR (K), 3-,and 6-month PFS (L-M) to correct publication bias using the trim and fill method.

**
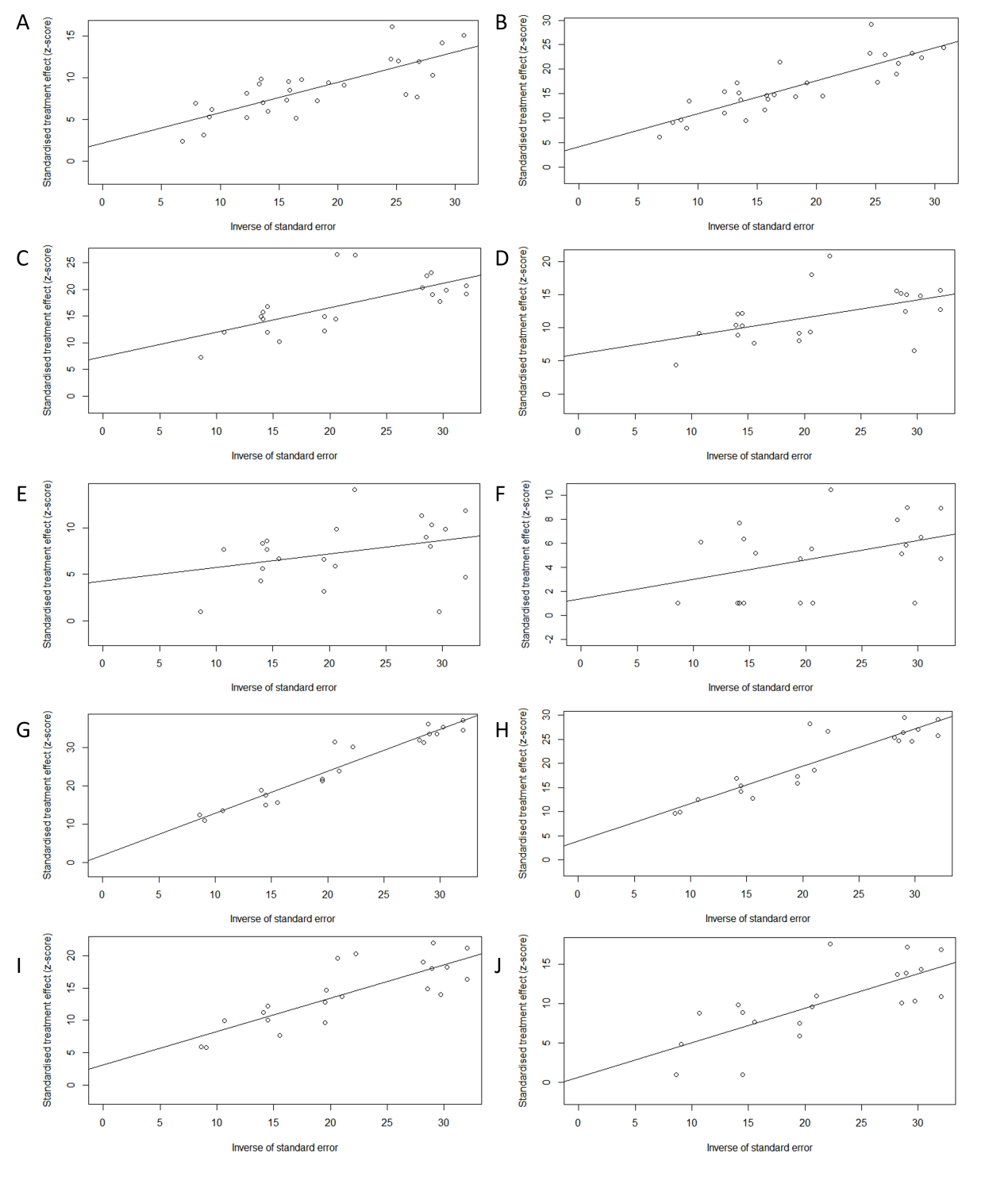
Supplementary Figure 2.** Egger tests of ORR (A, P=0.055), DCR (B, P=0.013), 3-, 6-, 12- and 18-month PFS rates (C-F, P=0.009, 0.018, 0.062, and 0.485, respectively), 3-, 6-, 12- and 18-month OS rates (G-J, P=0.245, 0.059, 0.126, and 0.761, respectively).

**
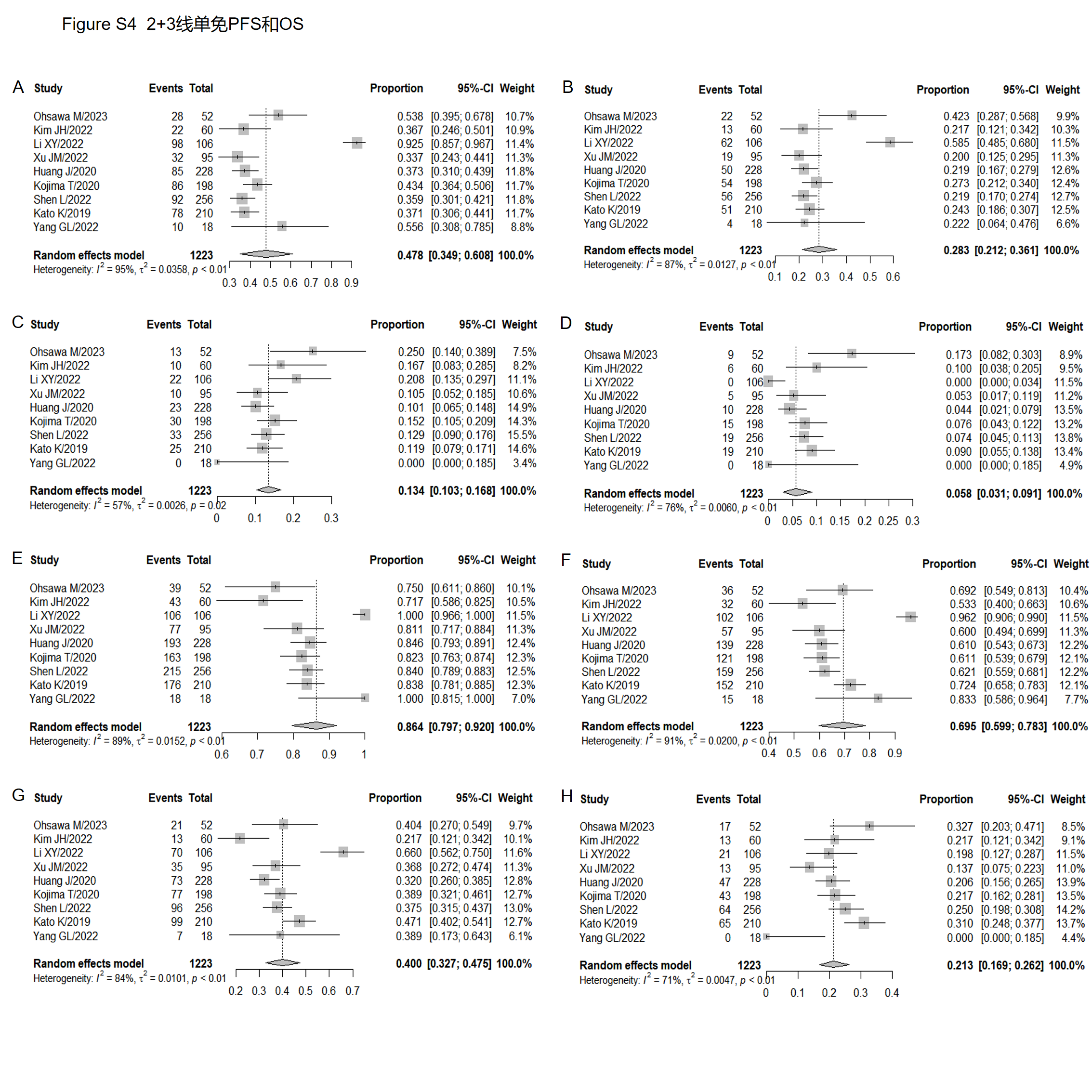
**

**Supplementary Figure 3.** Forest plots of 3-, 6-, 12- and 18-month PFS rates (A-D) and3-, 6-, 12- and 18-month OS rates (E-H) for patients treated with second or later-line PD-1 inhibitor monotherapy.

**
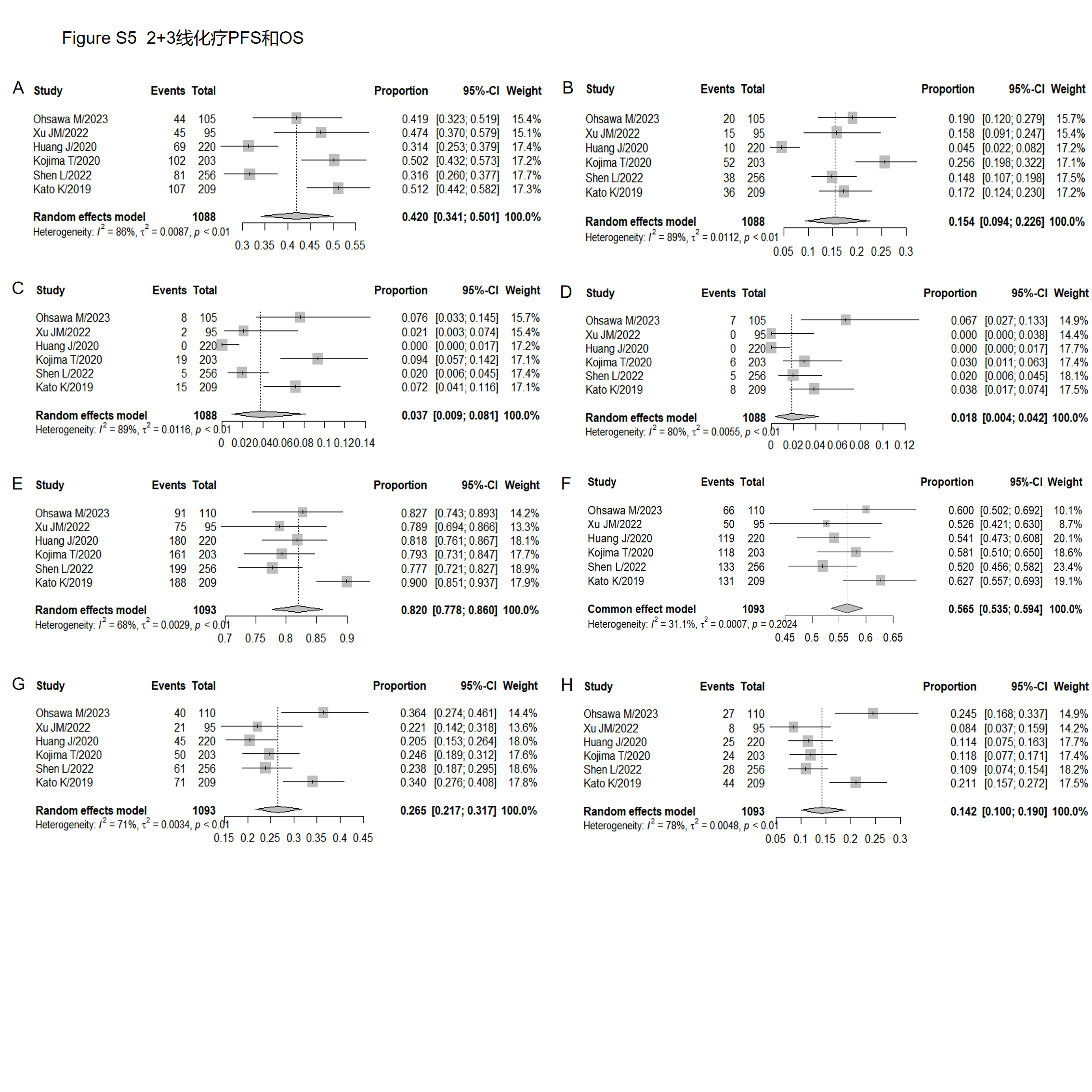
**

**Supplementary Figure 4.** Forest plots of 3-, 6-, 12- and 18-month PFS rates (A-D) and3-, 6-, 12- and 18-month OS rates (E-H) for patients treated with second or later-line chemotherapy.


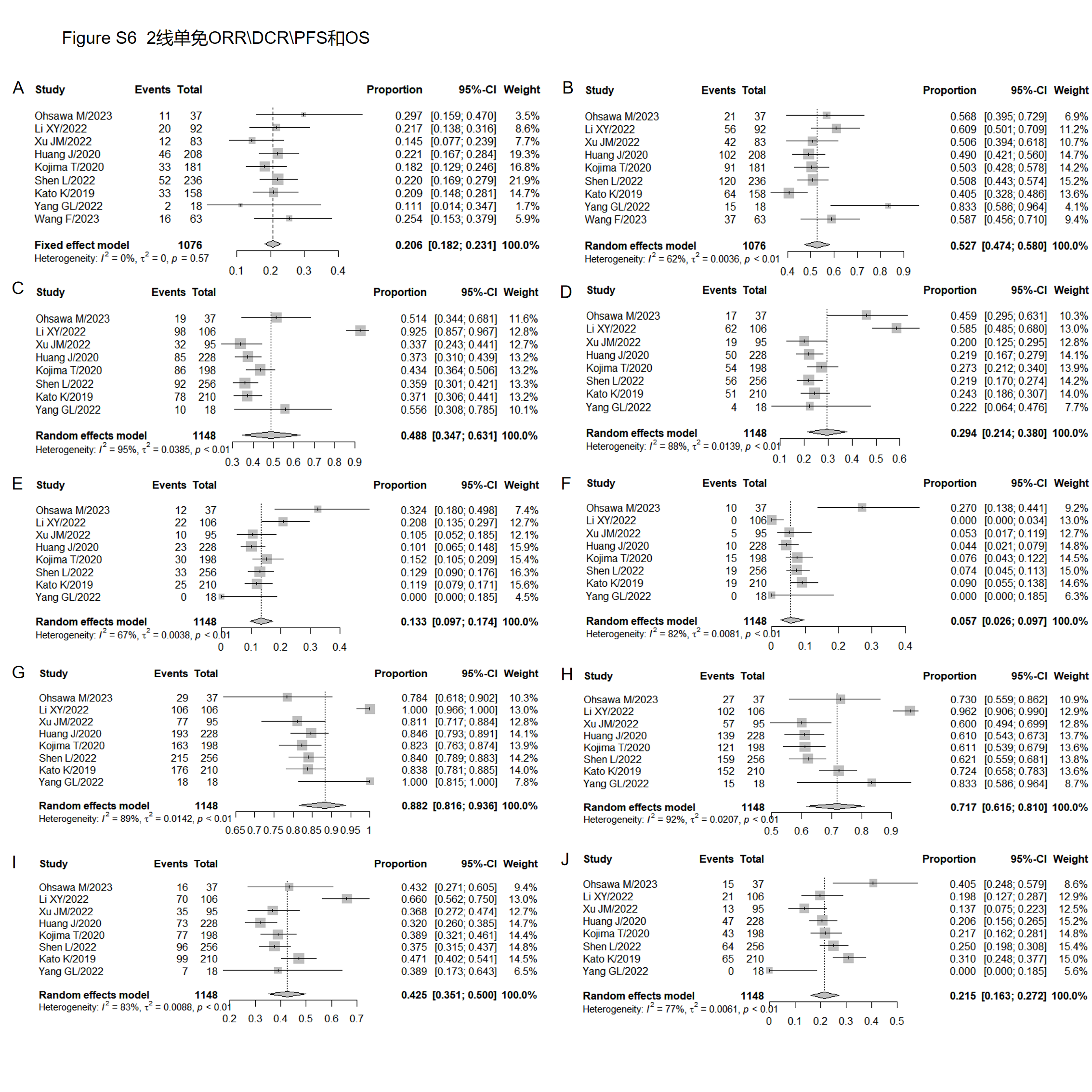
**Supplementary Figure 5.** Forest plots of ORR (A), DCR (B), 3-, 6-, 12- and 18-month PFS rates (C-F), 3-, 6-, 12- and 18-month OS rates (G-J) for patients treated with second-line PD-1 inhibitor monotherapy.


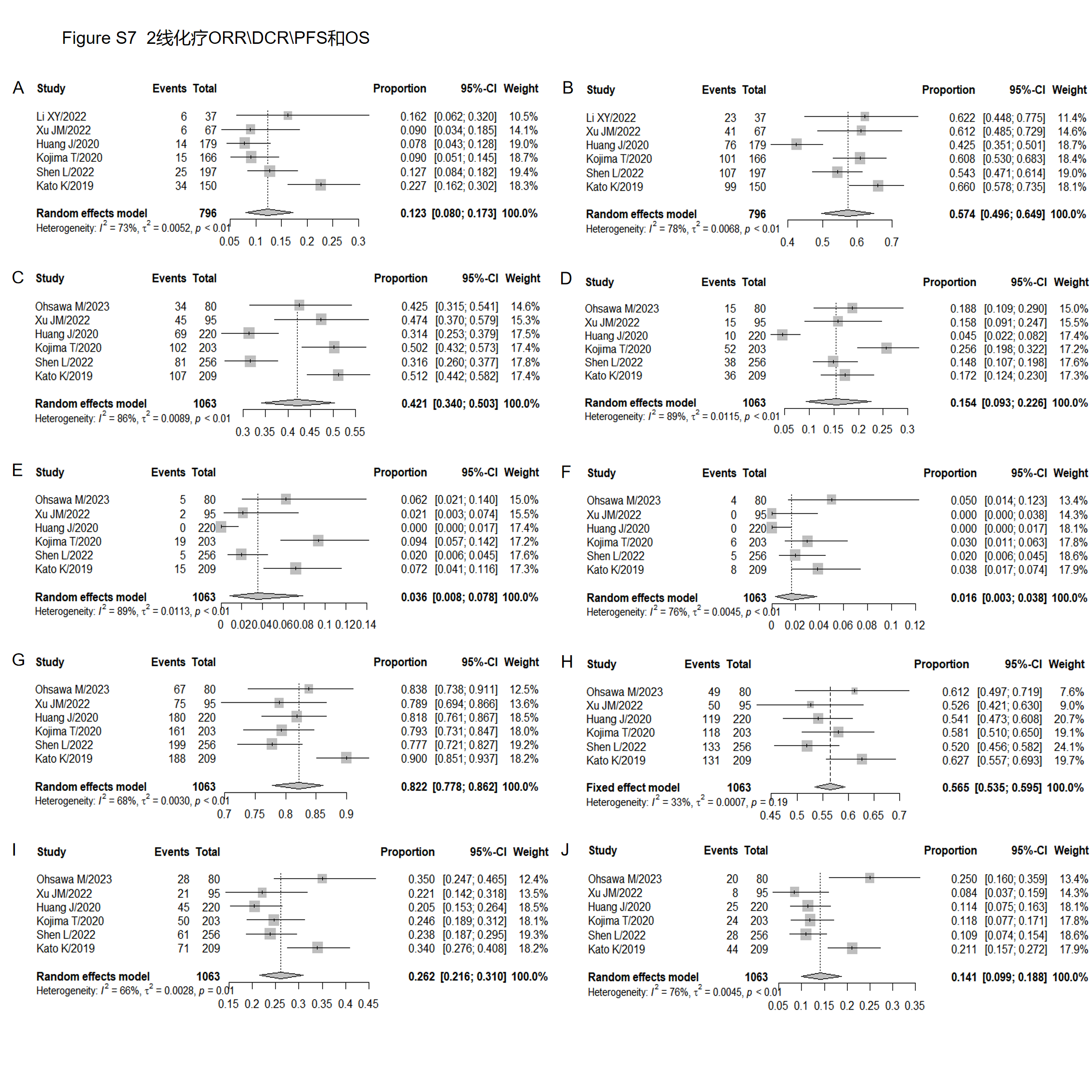
**Supplementary Figure 6.** Forest plots of ORR (A), DCR (B), 3-, 6-, 12- and 18-month PFS rates (C-F), 3-, 6-, 12- and 18-month OS rates (G-J) for patients treated with second-line chemotherapy.


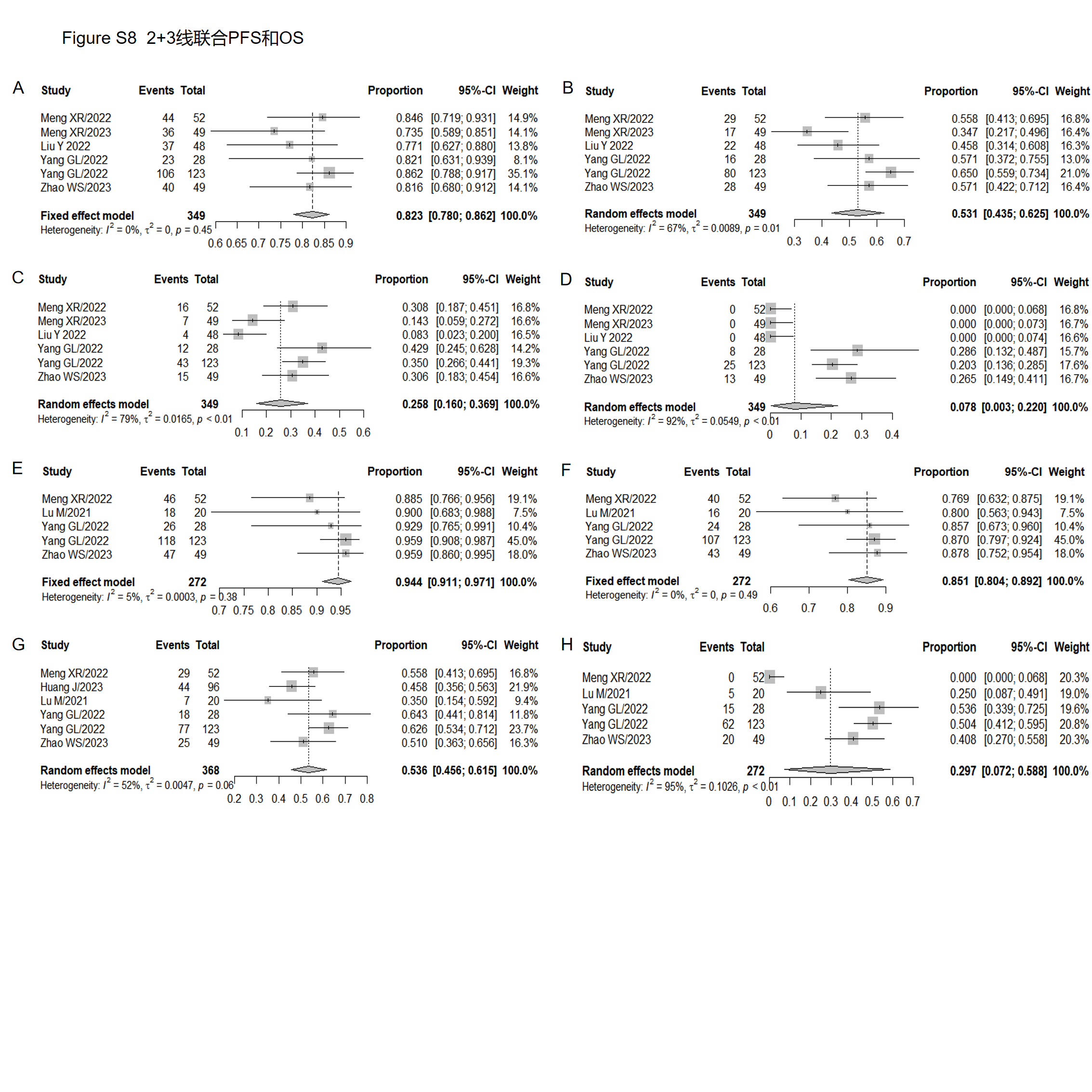

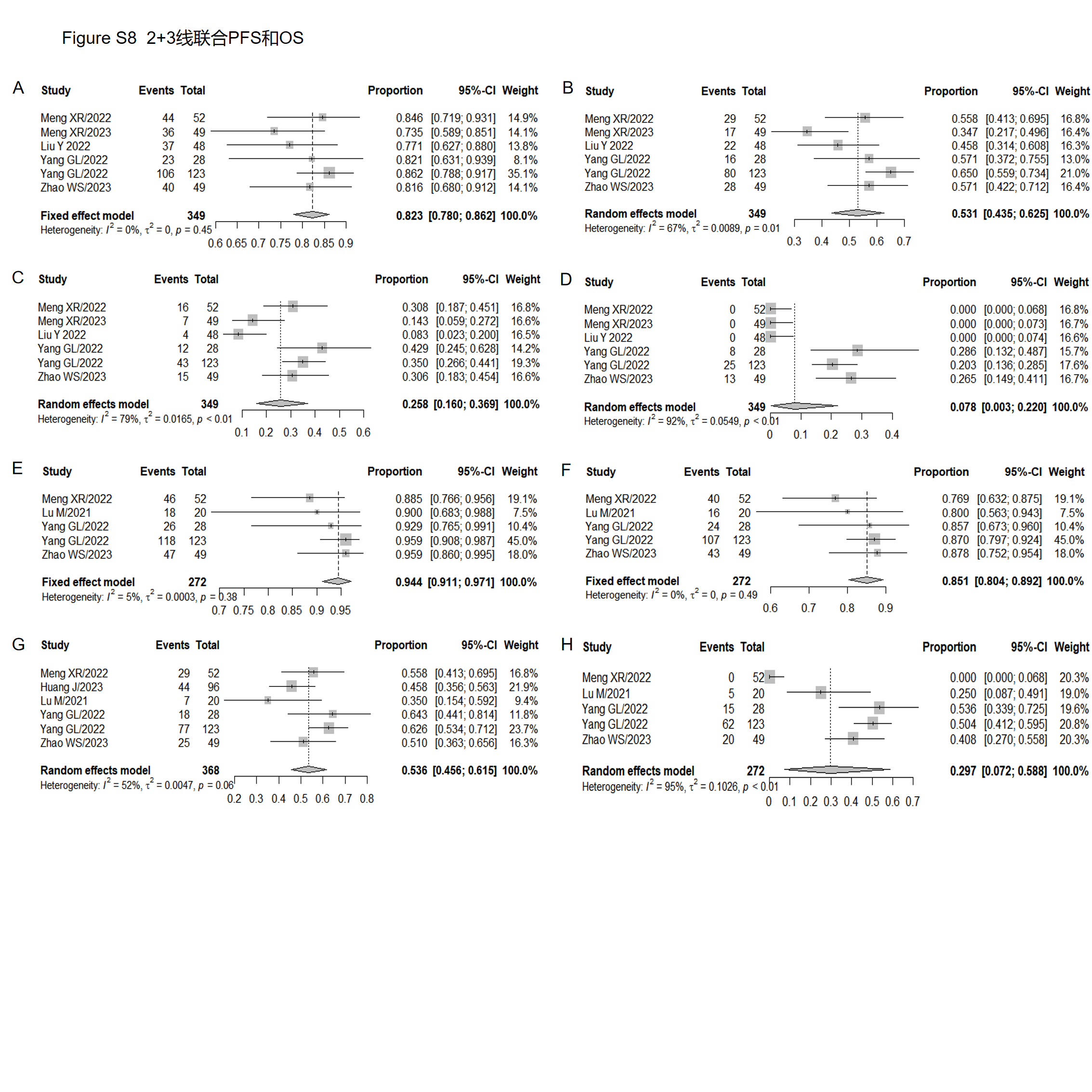
**Supplementary Figure 7.** Forest plots of 3-, 6-, 12- and 18-month PFS rates (A-D), 3-, 6-, 12- and 18-month OS rates (E-H) for patients treated with second or later-line combination immunotherapy.

**
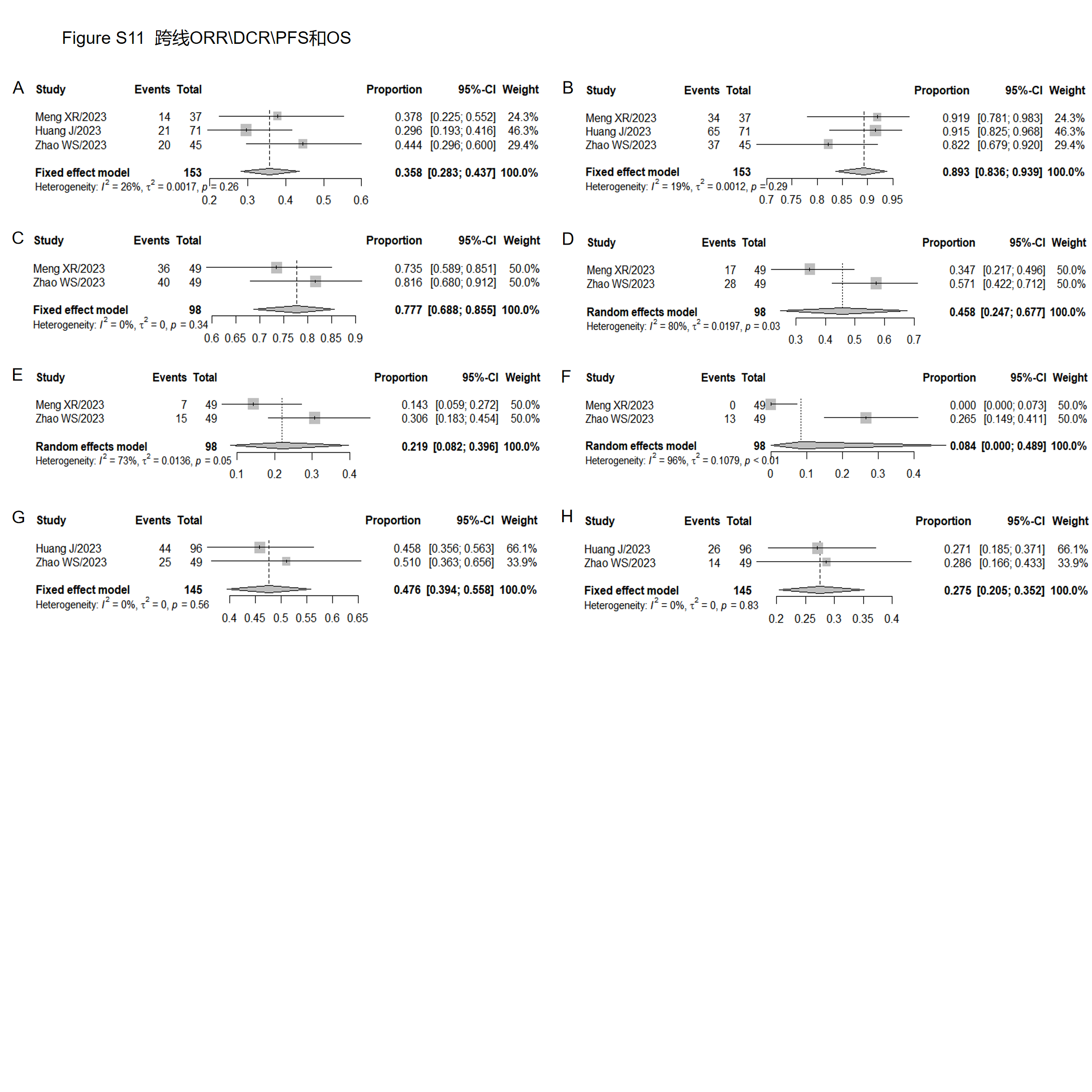
Supplementary Figure 8.** Forest plots of ORR (A), DCR (B), 3-, 6-, 12- and 18-month PFS rates (C-F), 12- and 24-month OS rates (G-H) for combination immunotherapy in immunochemotherapy-treated advanced ESCC patients.


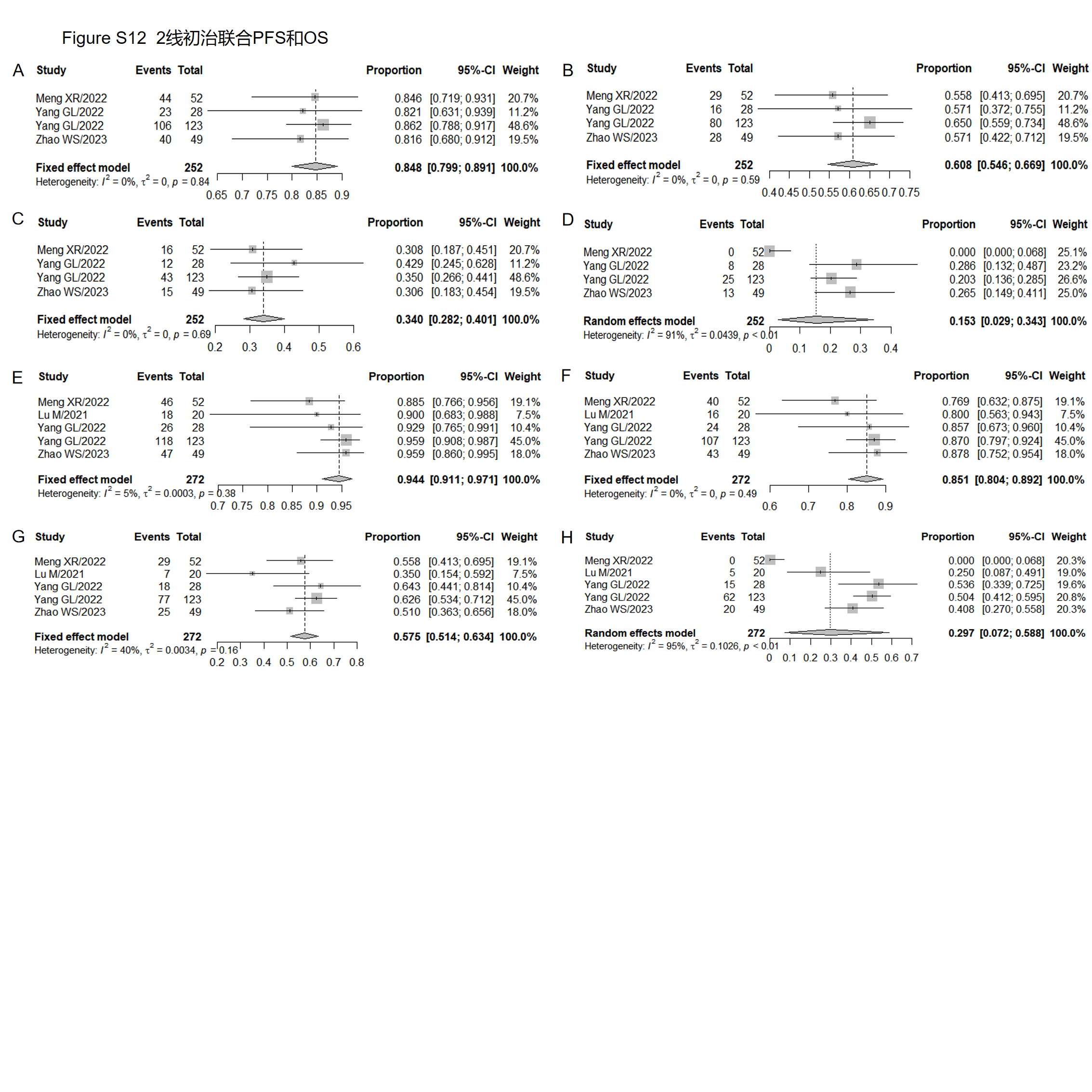
**Supplementary Figure 9.** Forest plots of 3-, 6-, 12- and 18-month PFS rates (A-D), 3-, 6-, 12- and 18-month OS rates (E-H) for immunotherapy-naive patients treated with second-line combination immunotherapy.

**
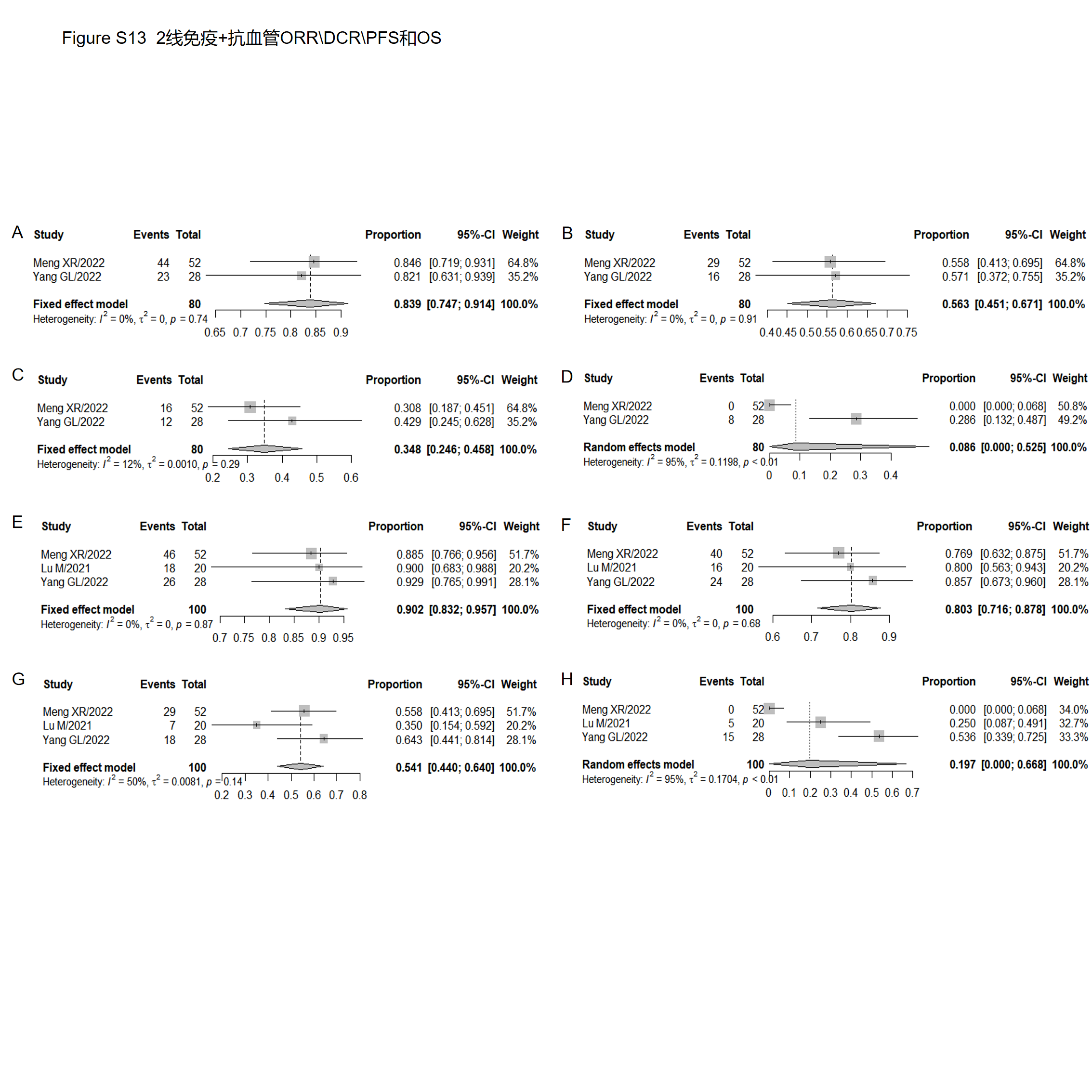
Supplementary Figure 10.** Forest plots of 3-, 6-, 12- and 18-month PFS rates (A-D), 3-, 6-, 12- and 18-month OS rates (E-H) for patients treated with second-line PD-1 inhibitor combined with anti-angiogenesis therapy.

**
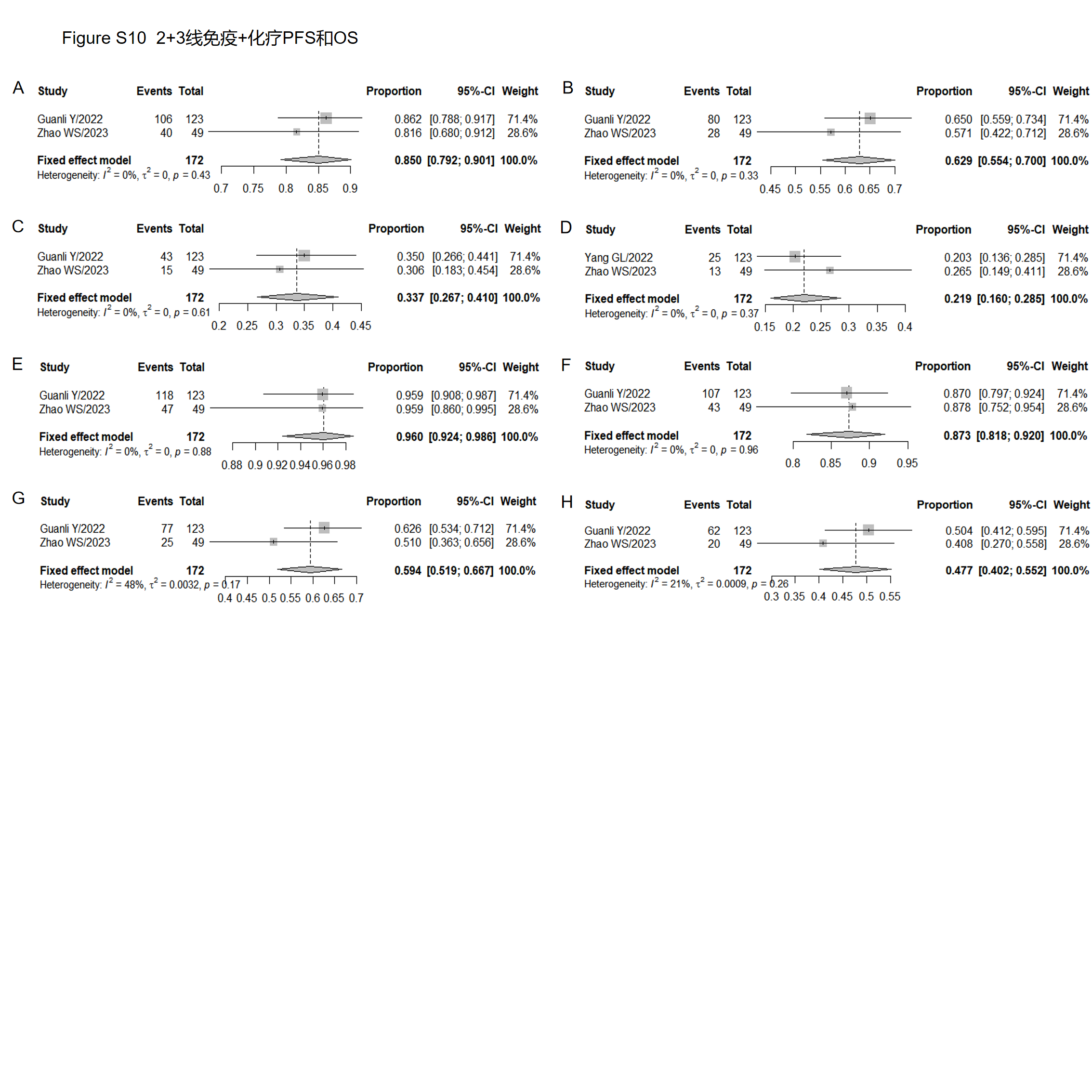
Supplementary Figure 11.** Forest plots of 3-, 6-, 12- and 18-month PFS rates (A-D), 3-, 6-, 12- and 18-month OS rates (E-H) for patients treated with second-line PD-1 inhibitor combined with chemotherapy.

**
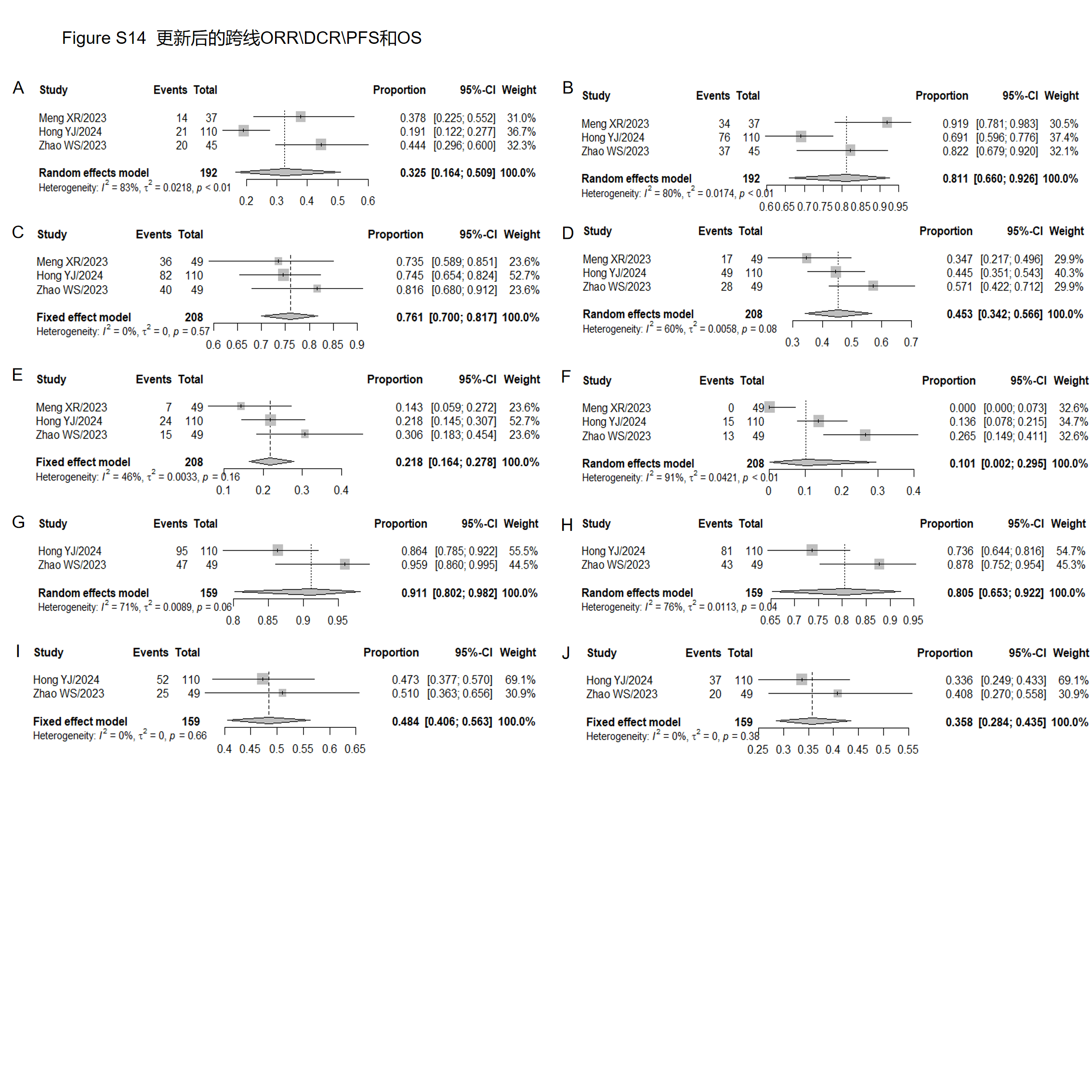
Supplementary Figure 12.** Updated forest plots of ORR (A), DCR (B), 3-, 6-, 12- and 18-month PFS rates (C-F), 3-, 6-, 12- and 18-month OS rates (G-J) for combination immunotherapy in immunochemotherapy-treated advanced ESCC patients.
